# Evaluating an MFI Community Health Worker Program: How microfinance group networks influence intervention outreach and impact

**DOI:** 10.7189/jogh.09.010435

**Published:** 2019-06

**Authors:** Roman Hoffmann

**Affiliations:** Wittgenstein Centre for Demography and Global Human Capital (IIASA, VID/ÖAW, WU), Vienna Institute of Demography / Austrian Academy of Sciences, Vienna, Austria

## Abstract

**Background:**

Community Health Workers (CHWs) are considered to be a cost-effective and inclusive solution to address the persistent health workforce shortage in many low and middle-income countries. In recent years, microfinance institutions (MFIs) got increasingly engaged in providing health services delivered by CHWs. Despite their growing importance, little is known about the impacts and implementation barriers of these mostly small-scale initiatives. This paper evaluates an MFI-led CHW program in the Philippines and studies the role of microfinance group networks in influencing program outreach and impact. The intervention aims at disseminating information in poor communities, improving health monitoring through increased check-ups and raising social support.

**Methods:**

Clustered randomized controlled trial in 70 communities in the greater area of Metro Manila, the Philippines. The main data was collected in a baseline and follow-up survey and is complemented with extensive sociometric network and geographical data. The main outcome variable is a composite health index based on 10 indicators. The role of the health worker’s embeddedness and connectedness in the community for program success is tested using tools of social network analysis.

**Results:**

The intervention led to a 3.8% (95% confidence interval (CI) = 1.3, 6.4) improvement in the composite health outcome. Effects across indicators are mixed and mainly driven by changes in immediate health monitoring behavior: The probability for routine examinations increased in the treatment group by 10.6% (95% CI = 3.2, 18.1), for regular blood pressure checks by 9.6% (95% CI = 3.3, 15.9), and for having access to a health care provider by 7.2% (95% CI = 0.93, 13.5). No statistical effects on general knowledge and social support are observable. Social networks are a key driver of program outreach and impact. Close friends and acquaintances of health workers used and benefited substantially more from the program than more distant ties.

**Conclusions:**

Despite the promising immediate behavioral impacts, it remains questionable to what extent such small-scale MFI initiatives can bring transformative and sustainable changes without external support. Microfinance group networks played an important role for the success of the health intervention and further research is needed to better understand how these affect the health care utilization decisions of the clients.

Several low- and middle-income countries face a considerable shortage of human resources in health care, often leaving the poorest parts of the population underserved. According to the WHO, in 2013, there was a lack of more than 7 million skilled health workers worldwide with countries in Sub-Saharan Africa and South- as well as Southeast Asia being particularly affected [1]. As a result of the shortage, millions of people are disabled or die every year from preventable and/or treatable diseases. The shortage has been acknowledged as a major constraint in building resilient health systems and in achieving health-related development goals on a global scale [2-4].

Community health workers (CHW) have been proposed as a cost-effective and inclusive solution to address the persistent shortage [5-8]. CHWs are members of the underserved communities who work as support health personnel in their neighborhoods after receiving a short health training. Among others, CHWs have been used to disseminate information, promote health care utilization and healthy lifestyles, monitor treatment compliance, and perform small routine check-ups. Because of their close relationship to the communities they can serve as a bridge between health providers, social and community services, and their peers in the neighborhood. This enables them not only to widen the supply of health services in the communities, but also to actively influence demand-side restrictions in health care utilization, such as informational or behavioral constraints [7,9-11].

This study evaluates a CHW program in the Philippines using a cluster randomized controlled trial design. The intervention was implemented by a microfinance institution (MFI), which traditionally provides access to financial services to the poor. Following an integrated development approach, an increasing number of MFIs have started in the recent years to also provide non-financial services to their clients and as part of this trend, several have established health programs including CHW components [12-14]. Despite their increasing importance and outreach, little is known about the impact of such privately organized health initiatives and the potential barriers faced by the implementing organizations. There is especially little evidence on the activities organized by small to medium scale MFIs, which make up a considerable share of the microfinance market [15].

As opposed to major national health programs, MFI health initiatives have limited access to resources and public support. As MFIs’ core competencies lie in other fields, they have to build up expertise and capacities in health care or partner with external organizations. Despite these challenges, there are also potential advantages to the provision of community health care services through MFIs [16]. With more than 200 million clients worldwide, most of them living below the poverty threshold, MFIs are able to effectively reach out to the most underserved population groups and address their specific health needs. Moreover, many MFIs rely on a dense grass-root infrastructure with strong peer support among clients and regular group meetings, providing a favorable environment for CHW activities.

Apart from assessing the overall impact of the CHW intervention as primary research objective, this study explores mechanisms contributing to impact heterogeneities and differential acceptance of the program in the neighborhoods. A particular focus is placed on the role of *social networks* in the microfinance groups, which are expected to play an important role in moderating the uptake of the intervention and its effects. Building on a rich literature on the relationship between social networks and health [17-20], this study is the first to systematically explore how social structures and the embeddedness of health workers in communities affect the success of a CHW intervention. The main data for this study was collected using face-to-face-interviews in two survey waves in February 2014 (n = 792) and April 2015 (n = 1064, including 36 CHWs), before and one year after the intervention. The sample for the interviews was drawn among the female clients of the partner organization from 70 randomly selected communities in Metro Manila and the adjacent province of Rizal. The quantitative analysis is complemented with insights from explorative semi-structured interviews with health workers and staff members.

The CHW intervention had an overall positive impact of 3.8% (95% CI = 1.3%, 6.4%) on a composite health index. The CHWs were particularly successful in improving health monitoring indicators. Despite these improvements, there was no significant impact on general health knowledge and social support, suggesting that the intervention may have not generated substantial and lasting impacts in the communities that go beyond immediate effects. Social networks in microfinance group were a key driver of program outreach and impact. Well-connected CHW significantly raised acceptance for the program among community members with the direct peers of the health workers benefiting over-proportionally from the intervention.

The remainder of the paper is structured as follows. The next two sections provide further information on the evaluated intervention and the study context and discuss the research design and measurement of key variables. The findings are presented in the “Results” Section and discussed in the final section of the paper. The main text is accompanied by a rich supplementary material presenting detailed background information on the study context and previous literature (Appendix S1 in **Online Supplementary Document**), the evaluation design and study protocol (Appendix S2 in **Online Supplementary Document**), and sensitivity analyses (Appendix S3 in **Online Supplementary Document**).

## BACKGROUND AND INTERVENTION DESCRIPTION

### Institutional setting

The evaluated CHW intervention was implemented by the Kasagana-Ka Development Center Inc. (KDCI), a Philippine non-governmental non-profit organization. KDCI operates in impoverished neighborhoods in Metro Manila and the surrounding provinces, where it uses microfinance services as primary instrument in its fight against poverty. The organization supports almost 30 000 poor clients in 23 field offices with microcredits using a co-maker-lending model. The main target group of the organization are women, who run micro-enterprises to support their families with an additional income.

The female clients are clustered in microfinance groups, referred to as centers, which represent the KDCI administrative units in the neighborhoods. Each center has a size of up to 42 members and is supervised by a so-called socioeconomic officer, who manages the center activities and maintains records. Clients of each center convene once a week to settle loan repayments and to discuss personal matters. Besides microfinance services, KDCI provides non-financial services to its members, including benefits related to education, shelter, access to social protection, and assistance during calamity and periods of distress.

Despite major improvements in population health in the past 40 years, the Philippines still face considerable health challenges (see Appendix S1.1 in **Online Supplementary Document**). Large parts of the population, especially among the poorest, do not have access to appropriate health care contributing to high levels of health inequality in the country. The health sector suffers from a shortage of medical staff, especially doctors and nurses, which is partly due to the massive emigration of health workers to other countries. Annually, about 17 000 to 22 000 health professionals emigrate to work outside the country [21] making the Philippines one of the leading exporters of human health resources worldwide [22]. Because of the shortage, many public health facilities are understaffed resulting in long waiting times and discomfort for patients, discouraging care-seeking behavior [23].

### Community health worker training intervention

As a reaction to the deficits in the public health system and to better serve the clients’ needs, KDCI started a health program in 2009 in cooperation with the Healthdev Institute, a Manila based NGO with focus on health support, education, and capacity building. As supportive element of the health program, KDCI trains CHWs in a four-day training, during which participants obtain basic knowledge about medical conditions, treatments, and prevention strategies. Furthermore, they learn how to perform routine examinations, such as blood pressure measurement. The trainings are conducted by trained nurses and midwifes together with experienced CHWs who have worked at least 2 years as part of the KDCI community health program.

Because of the mainly female client base of KDCI, the CHW training particularly addresses issues related to women’s health and empowerment. Women in the Philippines are confronted with specific health challenges and vulnerabilities that require adequate responses [24]. With 120 deaths per 100 000 live births in 2012, maternal mortality is still among the highest in the region and many women do not have sufficient access to reproductive health and birth control resulting in a high unmet need for family planning and high fertility rates [25]. In addition, due to financial constraints, female household members in the poor communities often delay or evade care-seeking in order to avoid being a financial burden to their families.

The evaluated CHW intervention aims at addressing these health challenges with the goal of achieving long-term improvements in the health situation of the clients and their families. The program has three *main objectives*, which form the focus of this evaluation: The program aims at (i) disseminating information among clients both specific to the KDCI health program (eg, information about check-ups) and in general (eg, information about disease prevention); (ii) improving health monitoring by carrying out small check-ups and by encouraging members to search for professional help; and (iii) raising social support by establishing the CHWs as primary contact persons in case of an emergency or a health concern. All of these objectives can be described as intermediate outcomes in the partner’s logical framework, which aims at improving the overall health situation in the neighborhoods.

Various micro-level studies from low- and middle-income countries have shown the potential of CHW interventions for various health outcomes (for reviews see [8,26-29]). While most evaluations are focused on large-scale national programs, there is little evidence on CHW programs implemented by MFIs outside the public health sector [11,13,16,30]. The KDCI health workers can be seen as a form of *community health promoters* whose main focus is the prevention of diseases by promoting and encouraging good health practices and disseminating information in their immediate environment. In contrast to large-scale national CHW programs, previous research has reported mixed effects of such small-scale community health promoter interventions [5,13,31,32]. Main challenges that were identified in the literature are lacking resources and funding for the initiatives, limited organizational capacities and expertise, insufficient anchoring of the interventions in the communities, lacking incentives for health workers, low levels of supervision and monitoring, insufficient motivation, patient overload, and missing support, among others [7,33,34]. As one of the first, this study empirically explores the role of the health worker’s embeddedness in the community as a factor in influencing program outreach and impact [35]. For this, rich social network data are collected among the clients allowing to determine the health worker’s position and relationships in the microfinance group networks of the KDCI centers.

### Details on procedures of the training intervention

The candidates for the training are selected by the KDCI socioeconomic officers. They are required to be KDCI clients for a sufficiently long amount of time (usually at least one year), to be in good standing with the organization, and respected by the other center members. Other than that, there are no pre-defined criteria for the selection of candidates. Since its implementation in 2011, more than 400 KDCI clients have been trained as CHWs (as of December 2015). The CHWs, who became part of this evaluation, share a similar demographic profile as the regular client population (see Table S1 in **Online Supplementary Document**). All trained CHWs are female. On average, they are slightly younger (2.43 years younger, *P* < 0.1, mean age = 43 years)) and better educated (1.185 years more schooling, *P* < 0.05, mean schooling = 10.8 years) than their peers.

For each neighborhood center, a maximum of one member is trained as CHW. Participants are charged with a small training fee of PHP 1500 ( ~ US$31.5). With this fee KDCI wants to ensure that only motivated clients become CHWs and that the program is financially self-sustainable. For the purpose of this evaluation, the training fee was reduced to PHP 750 ( ~ US$15.8) to ensure high compliance with the intervention in the treatment group centers. When asked for their rationale for participating in the training, the interviewed CHWs named reputational gains and a wish to serve the community as main reasons. Many of the interviewed health workers also mentioned personal motivations for their participation in the training, such as being better able to care for their families in general or a sick household member in particular as well as an ambition to learn more about health prevention and treatments.

After the training, the participants return to their communities where they are introduced to the other clients in their new function as health workers. The KDCI clients and their families are the main target group of the intervention. In principle, however, the health workers are also allowed to serve other households in the communities. The services are provided free of charge and the CHWs usually live in walking distance to all center members. The weekly center meetings play a key role for the activities of the health workers as they offer a well-suited platform for disseminating information, for performing small routine check-ups, and for giving advices and consultation to other center members.

As of now, the CHWs in the partner’s program are not incentivized for their engagement, which is not uncommon for small-scale CHW interventions [36,37]. However, there are plans by the organization to introduce a compensation scheme (eg, in form of loan repayment reductions) in the near future. Besides personal relationships between CHWs and public health workers in the communities, there is no formally and systematically established links between CHWs and the health care system in the neighborhoods. Yet, CHWs are encouraged to reach out to other health services providers and to refer clients if needed. KDCI encourages their health workers to take an active role, but apart from irregular spot checks there is only little monitoring and supervision of the CHW’s activities. Socioeconomic officers are expected to overview activities of the health workers in their communities, but this has not been rigorously implemented and there are no systematic monitoring guidelines and structures in place. About once a year the CHWs are invited to a general assembly, where they exchange experiences and receive further trainings.

## RESEARCH DESIGN AND METHODS

### Sampling and data collection

**Figure 1:**
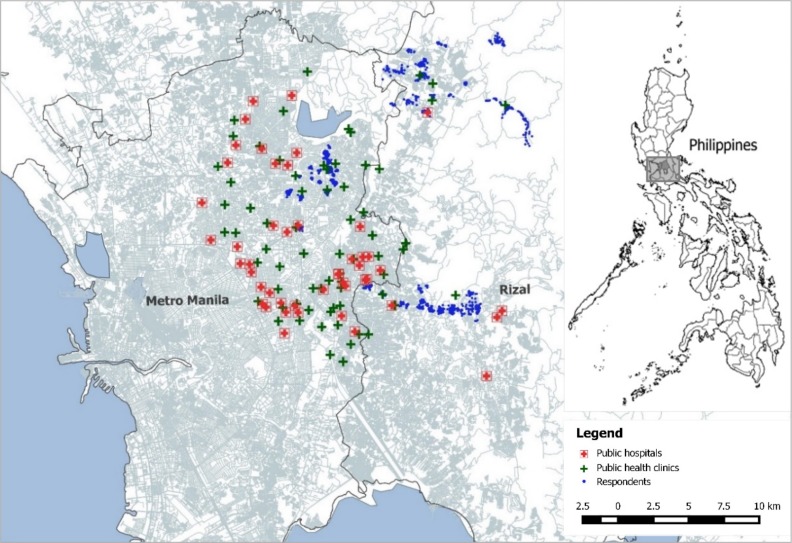
Map of study areas with locations of respondents’ homes and health facilities.

Three broad geographical areas in the north to northeast of Metro Manila were chosen as study areas. The selection was done based on two criteria: First, the selected study areas had to represent the diverse social and geographical background of the partner organization; and second, it was required that the CHW intervention was not yet implemented in most neighborhoods. The study areas cover both urban as well as rural populations with little access to health facilities (**Figure 1**).

In total, the three study areas encompass a population of more than 3000 KDCI clients in 142 centers. From within the three areas 70 KDCI centers with about 1500 clients were randomly drawn for the study. In the randomized controlled trial, the 70 KDCI centers in the sample represent the cluster units that were randomly assigned either to treatment or control group. The cluster randomization was stratified within the three study areas with half of the centers in each area being assigned to each treatment arm [38].

The data was collected in two survey waves in February 2014 and April 2015 covering a time span of more than a year. The *baseline survey* was conducted with a subsample of 792 respondents in February 2014. The intervention took place in April and May 2014 following the regular KDCI procedures. One year after the intervention, in April 2015, a *follow-up survey* was conducted to evaluate the impact of the CHW program. For this survey the sample size was increased by about 250 respondents, ie, in total 1064 persons participated in the follow-up survey. The final sample also included the community health workers to measure direct effects of the program on their health outcomes.

The evaluation uses primarily the follow-up data, as only a fraction of respondents participated in both survey waves. In addition to the main survey data, which was collected among the KDCI clients, semi-structured guided interviews were conducted with all CHWs as well as with selected staff members of the partnering organization The interviews were focused on the CHWs’ motivation to become a health worker, their daily routines, challenges they face in their activities, and the satisfaction with their work.

### Measurement

#### Evaluation outcomes and composite impact measures

**Figure 2:**
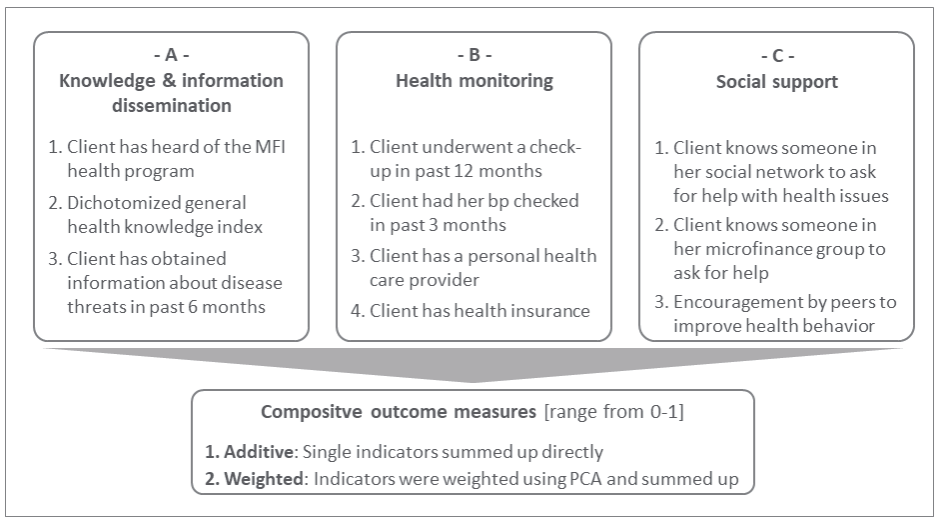
Outcome measures (all indicators binary coded).

We evaluate the impact of the CHW intervention along 3 outcome dimensions, which are based on the priority activities performed by the CHWs: (i) Dissemination of knowledge and information (3 indicators), (ii) improvement of health monitoring (4 indicators), and the (iii) raising of social support as primary contact persons (3 indicators). **Figure 2** provides an overview of the outcome measures. More detailed information on their measurement can be found in the supplementary material (Appendix S2.3 in **Online Supplementary Document**).

Each of the three categories was assessed with different indicators that were all binary coded except for two: A2 measures the general health knowledge of clients, which was derived from a knowledge index based on 28 questions about various health topics [39]. The measure was dichotomized at the median to make it comparable to the other binary measures. For C3, respondents were subsequently asked if someone among their friends or acquaintances criticizes them or gives them advices on their health behavior, actively shares information about health-related topics with them, encourages them to regularly undergo a routine check-up, and encourages them to seek help from a health professional if they are sick. The resulting encouragement indicator was coded one if respondents replied yes to at least 2 of the 4 sub-questions (median).

To study the overall impact of the intervention and to perform heterogeneity analyses, the 10 indicators were aggregated over the different dimensions using two procedures: Indicators were either summed up directly (additive approach) or weighted based on empirical weights calculated by employing principal component analysis [40]. Both the additive and weighted outcome measures were normalized to a range from 0 to 1.

#### Social network measures

As part of the main survey, respondents answered to a social network questionnaire with four questions about their relationship to all other center members. The resulting *sociometric data* provides not only information about individual relationships, but also about the structural position of clients in the entire microfinance group network of the center [41,42]. Respondents were asked who of the other microfinance group members they considered to be a personal friend, who they met at least once every week besides the regular center meetings, who they considered to be one of their five best friends, and to who they spoke about their personal, intimate problems.

Based on this information, it is possible to determine whether each client had a relationship with the CHW at the time of the survey. The relationships are classified in three categories: no relationship, weak relationship (personal friend or regular meeting), and strong relationship (best friends or speaking about personal, intimate problems). A relationship is assumed also for those connections which were not reciprocated by the CHW, or vice versa (asymmetric ties). In addition, the indegree centrality of the CHW is calculated [41,42]. The measure, which is normalized by the total center size, can be considered as an indicator for the CHW’s embeddedness or popularity in the center.

#### Control variables

To increase precision in the estimation, additional control variables are included in all models. Only such control variables are selected that cannot be influenced by the treatment to avoid estimation biases due to *bad controls* [43]. As additional socio-demographic variables the models control for personal education measured in years of education, age, household size, and marital status. Finally, dummies for the three wider geographical areas and measures for the existing health infrastructure in the neighborhoods – distance to the next health facility and the number of hospitals and clinics in a range of 2 and 5km around the respondent’s home – are included in the models.

### Identification strategy

In the empirical identification, the intervention impact is estimated by regressing the composite and individual outcomes on treatment status using ordinary least squares (OLS) and two-stage-least-squares (2SLS) estimation. The latter accounts for minor changes between treatment and control group centers (contamination of 3 centers <5% of all centers). For this, the actual treatment status is instrumented with the strictly exogenous original random assignment in a two-step procedure (see Appendix S2 in **Online Supplementary Document** in for first stage estimation and randomization/balance checks). Due to the changes in sample size between survey waves, the impact evaluation primarily relies on data from the follow-up survey (simple differences). Since the CHWs are themselves part of the sample, a dummy is included in the models, which indicates if the respondent is a CHW to control for differences in the outcome for the health workers.

The estimated impact represents *intention to treat effects* (ITE), ie, the effects of the intervention on all KDCI clients in the treatment group neighborhood, regardless of whether the clients made use of the CHW’s services or not [38]. For the partner organization the ITE estimate is the parameter of interest taking into consideration that there may always be some clients, who do not make use of the services provided by the CHWs. The second part of the evaluation deals with the actual mechanisms influencing program outreach and impact heterogeneities among the community members.

## RESULTS

### Descriptive statistics

**Table 1** shows summary statistics of the main outcome indicators across treatment and control group after accounting for contamination. The first set of binary coded indicators captures knowledge and information, the second health monitoring, and the third social support measures. The last two rows report the composite impact measures normalized to a range from 0 to 1. For most outcome dimensions, higher levels can be observed in treatment as compared to control group centers.

**Table 1:** Summary statistics of outcome indicators by treatment status*

	Actual treatment status					
**Outcomes**	**Control**		**Treatment**		**Total**	
**A. Knowledge/information dissemination**	**Mean**	**SD**	**Mean**	**SD**	**Mean**	**SD**
A.1 Informed about health program	0.61	0.49	0.69	0.46	0.65	0.48
A2. General health knowledge	0.54	0.50	0.53	0.50	0.54	0.50
A.3 Learning about disease threats	0.41	0.49	0.41	0.49	0.41	0.49
**B. Health monitoring:**						
B.1 Underwent check-up	0.40	0.49	0.48	0.50	0.44	0.50
B.2 BP measurement	0.30	0.46	0.36	0.48	0.34	0.47
B.3 Access to health care provider	0.57	0.50	0.65	0.48	0.61	0.49
B.4 Personal health insurance	0.20	0.40	0.22	0.41	0.21	0.41
**C. Social support:**						
C.1 Contact person in general	0.78	0.41	0.79	0.41	0.78	0.41
C.2 Contact person in center	0.86	0.34	0.88	0.33	0.87	0.34
C.3 Encouragement by peers	0.36	0.48	0.36	0.48	0.36	0.48
**Composite outcomes:**						
Additive	0.48	0.17	0.52	0.17	0.50	0.17
Weighted	0.44	0.21	0.48	0.21	0.47	0.21

SD – standard deviation

*Table displays the mean summary statistics for the outcome indicators used in the program evaluation.

**Figure 3:**
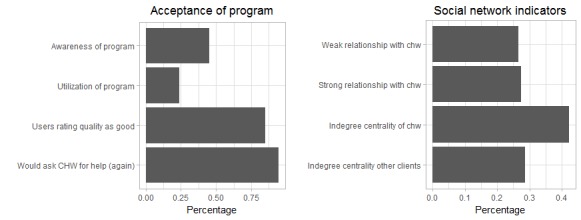
Summary statistics: program acceptance and social networks.

The acceptance of the program was limited among the KDCI client population (**Figure 3**). Only 44.8% of all respondents in treatment group centers were fully aware of the program, which was assessed with client’s ability to identify the CHW in their center by name, and only 23.3% said that they made use of the CHW services, eg, in form of consultations or minor check-ups (program uptake). Still, when asked how satisfied they were with the CHW’s services (scale from 0-10), the majority of users evaluated the CHW program with good grades (6-10) and said they would ask the CHW for help again if they had a health problem. However, there is considerable variation across centers. In 8 centers (22% of treatment group centers) users reported an average satisfaction level below 5.

The second part of the evaluation studies the role of social networks in influencing the acceptance and impact of the intervention in the treatment group centers. 26.6% of clients were weakly connected and 27.4% strongly connected with the CHW in their center. CHWs were on average better connected in the microfinance groups than their peers (indegree 0.42 vs 0.29). Again, some variation in the sample is observable with some CHWs being very well connected (max indegree of 0.875) and others not at all (min indegree of 0.043).

### Overall intervention impact

**Table 2** shows the differences in the additive and weighted composite health outcomes between treatment and control group centers. The intervention had a statistically significant positive effect on both outcome measures. According to the 2SLS estimation, the intervention led to an increase in the simple additive and weighted outcome by 3.8% and 3.6%, respectively. Compared to regular clients, also the CHWs had on average a higher value on both outcome measures. However, this effect is statistically not significant suggesting that – other than the difference resulting from being members of the intervention group centers – CHWs were not considerably different from regular clients. Regarding the controls, higher values of the composite outcomes are observable among the higher educated and respondents with higher cognitive abilities as assessed with a memory test. Similarly, older people showed higher values in the composite outcome, which may have resulted from their stronger need for health monitoring and greater knowledge about health issues.

**Table 2:** OLS and 2SLS estimation: Overall impact of the CHW intervention on composite outcome measures

	Additive outcome measure			Weighted outcome measure		
	**OLS**		**2SLS**	**OLS**		**2SLS**
Treatment group dummy	0.033**	0.038**		0.031*	0.036*	
	(0.012)	(0.013)		(0.013)	(0.014)	
CHW dummy	0.029	0.027		0.051	0.048	
	(0.030)	(0.030)		(0.036)	(0.035)	
Years of education	0.012***	0.012***		0.017***	0.017***	
	(0.002)	(0.002)		(0.002)	(0.002)	
Cognitive abilities	0.013**	0.013**		0.015**	0.015***	
	(0.004)	(0.004)		(0.004)	(0.004)	
Age	0.002**	0.002***		0.003***	0.003***	
	(0.001)	(0.001)		(0.001)	(0.001)	
Household size	-0.001	-0.001		-0.002	-0.002	
	(0.004)	(0.004)		(0.005)	(0.004)	
Number of children	0.006	0.006		0.010	0.010	
	(0.005)	(0.005)		(0.006)	(0.006)	
Marital status	0.015	0.015		0.019	0.020	
	(0.012)	(0.012)		(0.014)	(0.014)	
Distance to next health facility	-0.001	-0.001		-0.003	-0.003	
	(0.010)	(0.009)		(0.010)	(0.010)	
Number of hospitals in 2 km range	0.001	0.001		0.001	0.001	
	(0.003)	(0.003)		(0.003)	(0.003)	
Number of clinics in 2 km range	0.000	0.000		0.002	0.002	
	(0.004)	(0.004)		(0.004)	(0.004)	
Constant	0.233***	0.230***		0.144**	0.140**	
	(0.044)	(0.045)		(0.048)	(0.048)	
Observations	1057	1057		1057	1057	
Adjusted R^2^	0.054	0.054		0.081	0.080	
AIC	-633.42	-633.25		-399.82	-399.64	

OLS – ordinary least squares, 2SLS – two-stage least squares, AIC – Akaike Information Criterion

*OLS and 2SLS regression coefficients in cells, standard errors in parentheses. Standard errors are clustered on center level (m = 70). All models control for fixed effects of the wider geographical area.

*P*-value: * *P* ≤ 0.1, ** *P* ≤ 0.05, ***P ≤ 0.1.

### Intervention impact on separate indicators

**Figure 4:**
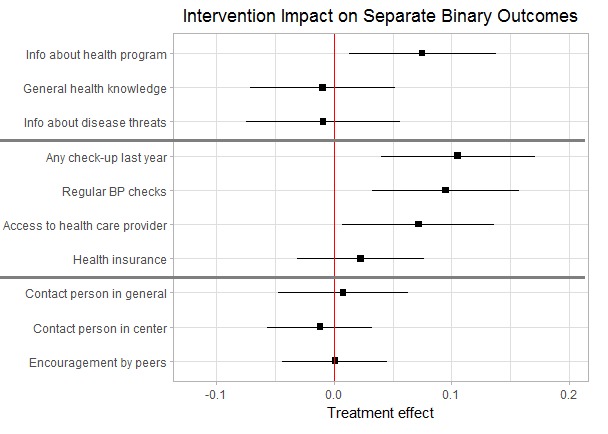
Intervention impact for separate binary outcome indicators.

**Figure 4** shows the intervention effects separately for the different outcome categories. The outcome indicators of each dimension are listed in the rows. The interpretation focusses on the 2SLS models, but all results are robust to the use of OLS. The CHW intervention had a significant positive effect on clients’ awareness of the KDCI health program and its services. According to the IV estimates, respondents from treatment group centers had a 7.5% (95% CI = 3.5, 14.9) higher probability of being aware of the KDCI health program. The more substantial general health knowledge indicators, on the other hand, were not significantly influenced by the intervention.

A positive impact is also observed for several of the considered health monitoring indicators, which can be directly performed by the CHW, such as bp measurement or small-routine check-ups. Respondents living in treatment group centers had a 10.6% (95% CI = 3.2, 18.1) higher probability of having undergone a check-up in the past year and a 9.5% (95% CI = 3.3, 15.9) higher probability of having had their blood pressure checked. Besides this, KDCI clients from treatment group centers reported a 7.2% (95% CI = 0.93, 13.5) higher probability of having access to a professional health care provider. Given the high disease prevalence in the neighborhoods and low levels of monitoring, these improvements can lead to substantial health gains. On the other hand, respondents from treatment group centers were not significantly more likely to have a personal health insurance.

With regard to social support, the intervention did not have a significant effect on any of the considered indicators. Respondents in treatment group centers were neither more likely to know someone in their social network or their KDCI center to ask for r help or advice nor more likely to be encouraged by their peers. Although the CHW intervention led to improved health monitoring, this seems to have not translated into an increase in perceived support in the communities. Apparently, the majority of clients did not perceive the community health workers as valuable support with their health issues suggesting that the program has not reached all of its priority objectives.

### Social influences on program outreach and impact

One reason for the restricted impact may be the overall low levels of program utilization and awareness among the wider client population. Indeed, additional analyses (Appendix S3.4 in **Online Supplementary Document**) show that a large share of the impact heterogeneity between neighborhoods are attributable to differences in program acceptance as measured with the clients’ utilization and awareness of the program.

In the final step of the evaluation, the role of the CHW’s embeddedness in the client network as determinant of program outreach and impact is studied. **Table 3** shows the results of logit and OLS models, which regress clients’ awareness and utilization of the program as well as the composite additive outcome on different network indicators. The unit of observation are the clients in the treatment group excluding the health workers (n = 538). In a first step, the effect of the overall indegree of the CHW (range 0-1) as a proxy for her status in the center is examined. In a second step, the models are extended by additionally including relational indicators measuring whether the individual client had a weak or strong relationship with the health worker to measure effects of direct connectedness.

**Table 3:** Logit and OLS models: Social network drivers of program acceptance and impact*

	Program awareness Logit		Program utilization Logit		Additive outcome OLS	
	**- 1a -**	**- 1b -**	**- 2a -**	**- 2b -**	**- 3a -**	**- 3b -**
**Social network indicators:**						
Indegree centrality of CHW [0-1]	0.561**	0.198	0.729***	0.357*	0.151*	0.068
	(0.208)	(0.222)	(0.151)	(0.155)	(0.064)	(0.071)
Weak relationship with CHW [0/1]		0.186***		0.159**		0.070**
		(0.050)		(0.050)		(0.024)
Strong relationship with CHW [0/1]		0.333***		0.354***		0.062*
		(0.051)		(0.029)		(0.026)
**Additional controls:**						
Network size	-0.002	0.001	-0.001	0.002	-0.002	-0.001
	(0.003)	(0.004)	(0.003)	(0.003)	(0.002)	(0.002)
Network density	-0.636	-0.273	-0.760*	-0.297	-0.360+	-0.300
	(0.390)	(0.395)	(0.308)	(0.329)	(0.193)	(0.184)
Geographical distance	-7.427	-6.068	-3.245	-2.932	2.802	3.261
	(5.272)	(5.732)	(6.574)	(5.820)	(1.899)	(2.024)
Observations	538	538	538	538	538	538
Pseudo/adjusted R^2^	538	538	538	538	0.018	0.043
AIC	0.032	0.091	0.083	0.219	-308.98	-320.86

CHW – community health worker, OLS – ordinary least square, AIC – Akaike Information Criterion

*Marginal effects and OLS coefficients in cells, standard errors in parentheses. Standard errors are clustered on center level (m = 37). All controls included in the models, but not displayed: years of education, cognitive abilities, age household size, number of children, marital status, neighborhood dummies, distance to next health facility, number of clinics and hospitals in 2 km range.

*P*-value: * *P* ≤ 0.1, ** *P* ≤ 0.05, ***P ≤ 0.1.

The models additionally control for characteristics of the microfinance groups, ie, the size and density of the networks, and the distance between the respondent’s and the health worker’s homes. The latter ensures that any social influence effects do not merely reflect geographical proximity. As the estimation is no longer based on an experimental identification strategy, I refrain from a causal interpretation of the results. Nevertheless, the findings are indicative for interesting relationships worth a further exploration.

When the effect of the indegree centrality is considered in isolation (models a), a clear positive relationship between the CHW’s status and the program’s outreach and impact is observable. A 10 percentage point increase in the CHW indegree, raises the clients’ program awareness by 5.61%, their utilization of the program’s services by 7.29% and the overall impact of the intervention by 15.1%. Once the individual relational indicators are controlled for (models b), the positive indegree effect is significantly reduced and becomes insignificant in two of the three models. This suggests that it is the direct connectivity of the CHW rather than her status, which drives the effects. Only for the case of the utilization of the services an independent status effect can be identified.

The relationship effects under control of the indegree and other network characteristics are substantial: While weak ties of the CHW are by 18.6% and 15.9% more likely to be aware of and utilize the intervention, being a close tie raises awareness and uptake even more substantially by 33.3% and 35.4%, respectively (significantly different from weak ties at 0.01). Interestingly, as additional analyses show, the social network effects are restricted to first order peers and do not affect more distant friends (eg, friends of friends of the CHW). This suggests that more far reaching social spill-overs, such as learning about the benefits of the intervention through friends, are limited in the studied microfinance group networks. Importantly, while there is a relationship between social connectedness and the outcomes, geographical connectedness, ie, distance to the CHW’s home, is insignificant further highlighting the importance of the social dimensions in influencing clients’ decisions.

**Figure 5:**
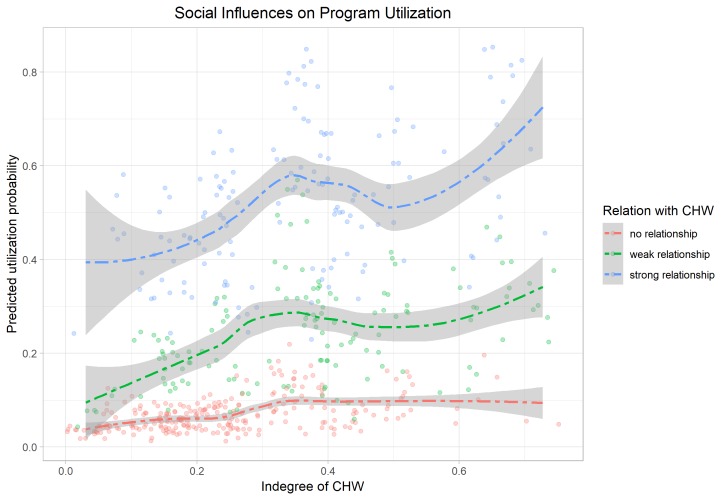
The role of network characteristics for program utilization.

**Figure 6:**
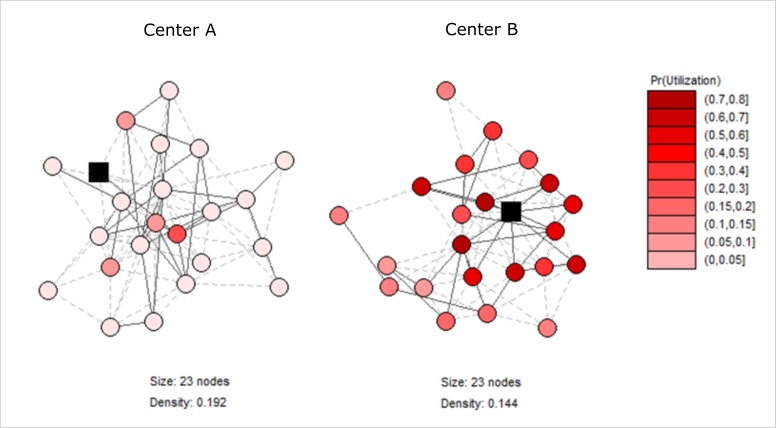
Exemplary microfinance group networks with different positions of the CHW.

The magnitudes of the social network effects are illustrated in **Figure 5** and **Figure 6**. The first graph shows the client’s probability to make use of the CHW’s services on the y-axis and the indegree of the CHW in her center on the x-axis. Splines are estimated and plotted separately for clients with no relationship (red), weak relationship (green), and strong relationship with the CHW (blue). Clearly, clients with a weak and strong relationship express a significantly higher utilization probability. The status effect seems to be moderated by the existence of a tie with the health worker suggesting that the health worker’s embeddedness or status in the network affects the client’s program uptake only if they are directly connected.

To further illustrate the relationships, **Figure 6** showcases two exemplary network graphs of microfinance groups of identical size and similar density, but with a different position (black squares) of the health worker in the networks. The coloring of the nodes, representing the clients in the group, shows the predicted utilization probability with darker red colors indicating higher values. Whereas the health worker takes a very central position in the right network, she is merely connected in the left network with clear consequences for the predicted take-up of the intervention.

The relational indicators are also strongly associated with the composite impact measures, which are analyzed in the final models 3. Respondents with a weak or strong tie to the CHW have a 7.0% and 6.2% higher impact than unrelated clients. These differences do not necessarily have to result from the higher awareness and uptake levels among directly connected clients, but could also reflect pre-treatment differences in the outcomes resulting from homophilous peer-group formation and selection effects [44]. To gain further insights in the direction of the relationships, additional tests are performed (see Appendix S3.6 in **Online Supplementary Document**): (i) 2SLS models are estimated instrumenting the relationship status with a plausibly exogenous measure of social distance. Confirming the previous findings, the models show a significant and robust effect of connectivity on the outreach and impact measures. (ii) Furthermore, using baseline survey data and comparing the pre-treatment level of selected outcome indicators no significant differences are observable between centers with a high and low status CHW and between clients with and without a connection to the health worker. This further suggests that the observed impact heterogeneities are in part driven by the social structures in the microfinance groups, which have a strong influence on whether clients are aware and make use of the CHW intervention.

## DISCUSSION AND CONCLUSION

Since its creation, KDCI has gradually extended its CHW initiative by allocating more resources and by stronger aligning the program to its core activities. The evaluation has shown that the program, which provides basic health care services to the clients, has an overall positive impact on health outcomes in the communities. The intervention led to improvements in specific health knowledge about the activities of the partner organization and health monitoring. While these effects may not generate immediate health gains, they can be seen as preconditions for reaching long-term improvements. Especially, given the low levels of monitoring and care-seeking and the high prevalence of infectious diseases with high external costs in the impoverished neighborhoods, the reported effects on health monitoring are expected to translate into more manifest health impacts in the long-run.

On the other hand, the intervention had no significant impact on other substantial and more lasting individual outcome categories, such as general health knowledge or social support. Although many respondents in the treatment group were aware of the existence of a CHW in their neighborhood, they seem to have not perceived them as particularly useful in supporting them with their health situation. The evidence on the impact of the intervention across outcome categories is hence mixed. This is in line with findings from other studies, which report limited effects of small-scale community health promoter interventions, suggesting that these programs alone may not be sufficient to bring transformative changes [8,13].

One explanation for the restricted impacts of the evaluated intervention are lacking interest among the client population and a low willingness to make use of the offered services. Exploring some of the underlying mechanisms influencing program outreach and impact, social networks were found to be an important driver. In particular, well-connected health workers were able to reach out to more clients with impact rates being higher among their immediate, ie, directly connected, peers. These findings encourage the targeting and selection of central actors in networks as health workers [17,45]. The findings are consistent with other work showing that strong ties are more influential than weak ties, especially in interactions related to intimate topics, such as personal health care [17,46-48].

While previous research was primarily focused on geographical proximity [49-51], this study emphasizes the important role of social proximity in influencing health care utilization decisions. Missing familiarity between the CHW and the community members may limit their trust in the health worker and their willingness to obtain support. Implementing organizations should ensure that CHW interventions are well aligned to the existing social structures in the neighborhoods. The organization’s ability to make effective use of the social capital inherent in the community networks and to mobilize community participation can largely influence program success [52].

Additional qualitative evidence from the semi-structured interviews with the health workers and the KDCI staff revealed different challenges that may have reduced the impact of the intervention. Some of the challenges are commonly found in voluntary or low-compensated health worker initiatives: The screening and selection of the health workers was carried out very unsystematically and may have not attracted the best candidates [53]; health worker retention and attrition was a problem [54]; there was a lack of continued support, supervision, and promotion of the health workers [55]; the link between the health workers and the public health care system was weak [56]; and beyond reputational gains as main motivator, clear incentives for the health workers were missing [57-59]. The interviews also revealed that some of the health workers were unsure about their roles and tasks and did not feel respected by their communities.

Improving the structure of the CHW intervention by taking appropriate measures can help counteracting some of the problems. Importantly, the health worker training should not only cover substantial content, but also didactical and social skills on how to disseminate information and how to pro-actively approach the community [28,60]. This could be helpful especially for those health workers without strong social ties in the local community network. As further potential measures, the WHO highlights in a recent review and guideline article, which summarizes experiences with CHW programs worldwide, the importance of comprehensive and systematic screening processes, competency-based formal certification, supportive supervision, clear and rewarding remuneration schemes, a strong integration of CHW programs in the existing health system, and the close involvement of the served communities [7]. By helping to overcome the reported challenges, these measures can raise the quality of the intervention and ultimately its impact on the target group.

This study faces some limitations. First, all presented outcome indicators rely on self-reported survey data, which may be prone to reporting and measurement errors. This issue was addressed by using multiple indicators for the different outcome dimensions. Second, respondents were interviewed only one year after the start of the intervention. This may be a time period too short to observe the long-term effects of the intervention on the health outcomes and to assess whether or not the program impact is sustainable in the long run. Third, the analysis of social networks influencing program acceptance is mainly explorative. The outcomes of the analysis may be influenced by omitted variables (eg, environmental conditions in the neighborhood) and should hence be treated with care. Nevertheless, the findings point towards interesting relationships, which may play an important role in influencing the interest in and uptake of health interventions in other settings. And fourth, as for most RCTs, the generalizability of the results is restricted to the local setting and context, which can be crucial in influencing intervention outcomes.

So far, only few studies have considered integrated CHW programs of MFIs, and further quantitative and qualitative research is needed to determine the potential contribution and particular implementation barriers of such initiatives. As the evaluation shows, MFIs can offer a suitable platform for the provision of essential health services in poor, medically underserved communities, in particular related to basic tasks, such as specific information dissemination and health monitoring [16,52]. Yet, it remains questionable to what extent such small-scale MFI health initiatives can generate substantial and long lasting impacts without additional external support. Importantly, as shown, the local context needs to be taken into consideration. “CHWs do not exist in a vacuum” [61], but are part of a complex social structure. Future research needs to address the social dimension of CHW activities as critical determinant of program outreach and success. Apart from the social dimension, also other environmental factors need to be taken into consideration. In this respect, it is particularly important to better understand how MFI-led interventions can be best linked to the public health system and how to make use of potential complementarities between different health care providers. The valuable experiences with this internvention can inform other organizations and provide useful lessons for the development of adequate health policies.

## Additional material

Online Supplementary Document
