# Supplementary material for: Evaluating an MFI Community Health Worker Program: How microfinance group networks influence intervention outreach and impact
Source: J Glob Health. 2019 Jun 15;9(1):010435. doi: 10.7189/jogh.09.010435 (PMC6571112; doi:10.7189/jogh.09.010435)
Supplement: Online Supplementary Document [file jogh-09-010435-s001.pdf]

J Glob Health 2019, 9: 010435

Supplementary Document: Evaluating an MFI Community Health Worker Program: How Microfinance Group Networks Influence Intervention Outreach and Impact

Content

S1. Study Context and Previous Literature ... 2
S1.1 Country Setting and Relevance ... 2
S1.2 MFI Delivered Health Care Services: Challenges and Advantages ... 3
S1.3 Community Health Worker Interventions in Historical Perspective ... 4
S1.4 Previous Literature ... 5
S2. Evaluation Design and Study Protocol ... 6
S2.1 Intervention Structure and CHW Demographic Profile ... 6
S2.2 Data collection ... 8
S2.3 Details on Measurement ... 9
S2.4 Details on Identification Strategy ... 10
S2.5 Ethical Considerations ... 12
S2.6 Power Analysis ... 13
S2.7 Randomization and Balance Check ... 13
S2.8 Details on Social Network Data Collection ... 17
S3. Sensitivity Analyses and Further Results ... 18
S3.1 Descriptives ... 18
S3.2 Impact Estimation for Separate Outcome Indicators ... 20
S3.3 Impact Heterogeneity across Treatment Group Centers: The Role of Acceptance ... 22
S3.4 Social Influence: Full Models ... 24
S3.5 Sensitivity Checks ... 25
S3.6 Instrumenting Relationship Status with Social Distance Measures ... 26
S3.7 Differences in Outcome Variables by CHW status ... 28
References ... 29

S1. Study Context and Previous Literature

S1.1 Country Setting and Relevance

Despite being one of the fastest growing economies in Southeast Asia, poverty is still widespread in the Philippines with about one in fifth Filipinos living below the national poverty line [1]. The metropolitan area of Manila, which is the focal location of this study, has seen a remarkable population rise in the past decades due to a large inflow of migrants from rural areas. The high population pressure resulted in various urban challenges linked to poverty, such as lacking adequate habitation with a large percentage of the population living in danger areas, limited employment opportunities, poor water and sanitation management, and lacking access to basic urban services, including health services.

In the past decades, the Philippine government has started several reforms to achieve universal health care [2]. The reforms have addressed deficits in the public health sector, such as poor accessibility and insufficient financial support for medical treatments. As a consequence of these efforts, there have been significant improvements in the health situation of many families. Infant mortality (<1 year) and child mortality (<5 years) dropped between 1990 and 2012 by 58.8% and 62.8%, respectively. Maternal mortality decreased in the same time period from 209 to 120 cases (42.6%) per 100,000 live births. Overall, the life expectancy in the population increased from an average of 56 years in 1970 to over 70 years in 2015 [3].

Despite these progresses, significant challenges remain: The country lags behind most neighboring countries in terms of health outcomes and the achieved improvements often did not reach the poorest parts of the population contributing to high levels of health inequality in the country (both along social and geographical strata). This is also reflected in the mortality statistics of the country: For the year 2011 the World Health Organization reports a mortality rate of 25.6% probability for men and 13.7% probability for women to die between the age of 15 and 60, as compared to an average of 11.8% for men and 8.1% for women in the entire Western Pacific Region [4]. Also, health insurance coverage remains low, especially among the poorest households.

In many impoverished communities, access to health care and treatment is insufficient resulting in a large percentage of diseases not being properly treated and sometimes not even diagnosed [5,6]. The low health care capacities are partly due to the massive emigration of health workers. Annually, about 17,000 to 22,000 health professionals emigrate to work outside the country [7] making the Philippines one of the leading exporters of human health resources worldwide [8]. The government in the Philippines has reacted to the situation by advancing several reforms to strengthen universal health care

and to develop a decentralized primary health sector with a focus on community health care solutions [2].

S1.2 MFI Delivered Health Care Services: Challenges and Advantages

In recent years, an increasing number of MFIs got engaged in the provision of complementary health services. As the MFIs' know-how and structures are focused primarily on financial service provision, they may face significant organizational challenges as well as capacity constraints when widening their traditional portfolios. Yet, at the same time, there are unique advantages to the provision of health services through MFIs given their increasing number and widespread geographical presence [9,10]. MFIs serve more than 200 million borrowers of which a large share lives in severe poverty [11]. Building on a well-developed grass-root infrastructure both in urban and rural settings with close connections to the local communities, MFIs can effectively reach out to these mostly underserved populations and address their specific health needs.

MFIs may represent a well-suited distribution channel for health services not only because of their wide outreach, but also because most of them target women as main client group (84% of borrowers were female in 2016, Microfinance Barometer 2017). This is based on the view that women are more likely than men to reinvest their income to improve the living situation of their families in general and their children in particular, including investments in health [12,13]. Empowering women through microfinance and improving their access to health services and information may hence generate important spill-overs that benefit not only the women, but the entire household.

Moreover, the principles of microfinance heavily rely on group solidarity, trust, regular meetings, and common support, which matches the fundamental peer support idea of the CHW concept. Indeed, there is evidence showing that microfinance programs can create non-monetary effects on health by making households more resilient to health shocks through income smoothing opportunities [14], by reducing emotional stress, and by improving uptake of costly health services [15]. Yet, there is little rigorous evidence on the impacts of MFI-led CHW programs and the specific barriers faced by the implementing organizations [16].

Apart from their social mission, there is an economic rationale for MFIs to provide health-related services: Health shocks represent a major threat for the income earning activities of poor households and can result in loan defaults. By improving the health of their clients and by making them more resilient to health shocks, MFIs can increase their clients' productivity and hence reduce risks in their portfolios. At the same time, by offering additional services, the MFIs increase their attractiveness for new clients.

S1.3 Community Health Worker Interventions in Historical Perspective

Historically, the CHW concept is not a new one. The first larger programs were implemented in the 1950s and 1960s in East- and South-East-Asia. Among these, the Chinese Barefoot Doctor or Village Doctor Program, fully established in the early 1950s, was one of the most prominent which served as a guiding concept for many other initiatives, such as in India or Indonesia that soon followed. Until the 1970s more than 1 million Barefoot Doctors were trained and worked mostly in the underserved rural areas in the western parts of the country.

In 1978 the international community acknowledged the role of community participation in primary health care (PHC) in the *Alma-Ata Declaration*, in which the participating countries agreed that the "people have the right and duty to participate individually and collectively in the planning and implementation of their health care" (WHO 1978, 2). In the aftermath of the declaration, several countries started developing own public CHW programs.[1] Yet, only few years after the declaration in the late 1980s, negative experiences with large-scale programs and the debt-crises in several low-income countries led to a decrease in financial and political support and ultimately to a loss of momentum of the community participatory movement in PHC.

Only the 1990s and 2000s brought a revival of CHW programs due to the experiences with the global HIV/AIDS epidemic as well as the increasingly pressing shortage in professional health workers [18]. In particular, following the declaration of the Millennium Development Goals, international organizations have been actively promoting the use of lay health workers as an integral part of the PHC workforce and as a mean to achieve the health-related development goals on a global scale. The 1 Million Community Health Worker Campaign, for example, is a private-public partnership of UN agencies, civil society organizations, firms, and academia, which support the scaling-up of CHW programs worldwide.

In the past 50 years, non-governmental organizations (NGOs) played an important role in the development of the community participatory approach. In an attempt to extend health care to underprivileged populations not reached by the public sector, NGOs established CHW programs in parallel to and sometimes anteceding national programs.[2] Increasingly in the past years, also microfinance institutions (MFIs) got engaged in the provision of essential health services through CHWs. One of the first large-scale MFIs which started an integrated health program with a CHW component was BRAC (formerly the Bangladesh Rural Advancement Committee), a non-profit organization of Bangladeshi origin which operates in more than 11 countries today. As part of its program, BRAC has trained

---

[1] CHW programs were also established in several high income countries. For example, there are major CHW programs for disadvantaged populations, such as immigrants or seasonal workers, in the US. [60,61]

[2] Longtime, CHW were not only seen as providers of health care, but also as agents of change. Based on the ideals of participation and ownership, these were supposed to empower their communities and to increase self-reliance [62].

thousands of CHWs since the mid-1980s [19]. The majority of their so called frontline CHWs are members of one of BRAC's microcredit saving groups. Unlike the BRAC program, most MFI-led CHW programs today are of small-scale with restricted access to financial and human resources making it a challenge to successfully implement and operate these initiatives [20].

S1.4 Previous Literature

CHWs are active worldwide both in developing and developed countries. Major programs can be found in Brazil (Cufino Svitone et al. 2000), Pakistan [22,23], and India [22]. The CHW concept strongly builds on the idea of peer support in health care and prevention. Being themselves part of the underserved communities, the CHWs can effectively reach out to other community members providing not only health advice and consultation, but also emotional support and practical assistance [18,24].

Various micro-level studies from low- and middle-income countries have shown the potential of such peer interventions also reflecting the diversity of issues addressed (for an overview see Perry et al.(2014)). For instance, Davis et al. (2013) find a one-third reduction of childhood undernutrition in Mozambique after the introduction of a CHW program and Bhutta et al. (2010) finds positive effects on neonatal mortality and stillbirths in Pakistan. Further positive impacts of CHW programs have, among others, been reported for the distribution of oral rehydration salts and nutrients [27], the promotion of handwashing and clean environments [28,29], community education about the risks of Malaria, and the promotion and monitoring of antiretroviral therapies and tuberculosis treatments [30–32].

While most evaluations are focused on large-scale public programs, there is little evidence on CHW programs implemented by MFIs outside the public sector, which are often extensions of more conventional group health education programs (for an overview of evaluations on integrated health programs, see Leatherman et al. (2012), Geissler & Leatherman (2015), and Dunford (2001)). Armin et al. (2001) focuses on a reproductive health program offered by CHWs to microfinance clients in Bangladesh. Few months after program implementation, the authors find a significant increase in contraceptive use and a decline in fertility in the surveyed communities. Using a difference-difference-approach, Ahmed et al. (2006) find a BRAC community volunteer program for ultra-poor households to have large-scale positive effects on poverty and health-seeking behavior at formal institutions aligned with a reduction in self-care/self-treatment (see also Hadi (2002)).

Desai & Tarozzi (2011) represent one of the few rigorous studies on MFI-led CHW programs outside Bangladesh. The authors conducted a randomized controlled trial on the impact of microcredit and family planning services provided by CHWs in rural areas of Ethiopia. Interestingly, extending previous research, the authors also study complementarities between the provision of microcredit and

the health intervention (see also Dohn et al. 2004). They do neither find evidence for a positive effect of the two isolated interventions on contraceptive use, nor for potential complementarities. Women's preference for contraceptive methods other than the ones offered by the CHWs and norms enforced by the social networks may have contributed to an attenuation of the impact of the intervention.

Social networks have been shown to play an important role in influencing health and health care utilization [38–40]. Peers can influence the uptake of health interventions in various ways. They can be a valuable source of information, serve as role models, or impose normative pressures influencing individual health decisions (Fisher et al. 2014; Devillanova 2008; Jackson 2011). Also for CHW interventions they are of great relevance, as these programs strongly build on the idea of peer support and mutual trust in communities. To the best of my knowledge, there is no other study considering the influence of a lay health worker's embeddedness in a community on the utilization of her services [43]. Also, there is very little understanding of how microfinance groups influence clients' behavior in general, and health decisions in particular (Banerjee et al. 2013).

Previous research has highlighted the importance of an individual's structural positions in network as a main determinant of her ability to influence others in the network. A central position in the network, i.e. several ties to other network members, is often interpreted as a sign of social prestige, popularity, and status [45,46]. Outreach and impact levels are expected to be higher in those networks, in which the CHW takes an overall more central position. At the same time, being directly connected with the CHW may affect client's awareness and willingness to utilize the health worker's services. This effect is expected to be more pronounced, if the relationship is stronger. Health is an intimate topic and people with a closer relationship to the health worker are likely to have better access to her services and more trust in her abilities and discretion, raising their acceptance levels. Indeed, studies have found close ties to be more influential than weak ties in influencing health-related decisions [47–49]. (see also Prost et al. 2013; Fottrell et al. 2013; Saha & Annear 2014).

S2. Evaluation Design and Study Protocol

S2.1 Intervention Structure and CHW Demographic Profile

The evaluated program has three *main objectives*, which form the focus of this evaluation: The program aims at (i) disseminating information among clients; (ii) improving health monitoring; and (iii) raising social support by establishing the CHWs as primary contact persons in case of an emergency or a health concern. All of these objectives can be described as intermediate outcomes in my partner's logical framework. As long term goal, the aim is to improve the overall health situation of the poor by increasing monitoring activities and disease prevention, which are still underdeveloped in the com-

munities. It is worth noting that the different evaluated outcomes are closely related and may influence each other. For instance, by providing additional information about disease threats, CHWs can motivate their peers to undergo a check-up.

The KDCI CHW training sessions consist of 6 modules which cover the following topics: Functions and conduct of CHWs, the importance of good health for well-being, infection channels, the health system and primary health care in the Philippines, referral channels, the importance of a clean environment for healthy living, blood pressure measurement, right nutrition and its impact on health, the human body, vital signs, common diseases and their symptoms, disease prevention, antibiotics and the risks of self-medication, reproductive health and sexual transmittable diseases, ageing and female health, infant and child health, legal aspects of health, and female empowerment.

The CHW intervention took place in April and May 2014. It followed the regular KDCI procedures: (i) The CHW training was publicly announced to the center members. (ii) An interested member from each center was selected for the training. The selected person had to be a KDCI client for more than one year and had to be reliable, in good standing, and respected by the other center members. Usually, only very few clients volunteer to become a health worker, which often restricts the set of potential candidates. CHWs are trained only in larger centers (with a size of 20 clients and more), but this guideline is not strictly followed. (iii) After the first contact the selected person was given time to discuss the matter with her family. (iv) Upon agreement the client was invited to the training[3]. In total, 37 new CHWs (36 after accounting for one drop-out) were trained and started working in their communities.

There is no uniform definition of the CHW concept and functions. It is possible, however, to distinguish the programs along several dimensions: Apart from the distinction between public and privately organized initiatives, one can distinguish specialist and generalist CHW programs, whereby the former are directed towards a single health issue in the communities, such as tuberculosis or diabetes care. Perry et al. (2014) names four tentative types of CHWs: (i) Semi-professional auxiliary health workers (AHW) with extensive training for at least one year and a strong integration in the formal public health sector; (ii) health extension workers with extensive training of up to one year, who receive like the AHW a regular salary and have a fix working schedule; (iii) regular community health volunteers with specified duties, such as regular health monitoring, but a shorter training period; and finally, (iv) intermittent community health volunteers, who receive only a short orientation training and who mostly perform health promotion, information dissemination, and community mobilization activities. According to this typology, this evaluation studies a small-scale intermittent community health volunteer or promoter program in this evaluation.

[3] In two cases, the selected client refused to be trained as CHW. Following the regular procedures, the SOs selected another member as substitute, who later got trained as CHW in the center.

Table S1 shows the demographic profile of the CHWs compared to regular clients (based on data from follow-up surveys). On average, the community health workers, who were trained as part of this evaluation, are 42.8 years old (2.43 years younger than the regular client population) and have 10.8 years of schooling (1.19 years more than the regular client population). They live in households of about 5.25 members and 69% of them are married. On a scale from 0-10, they rate their subjective health with 7.44. In terms of their wealth, CHWs are not statistically different from other clients. On average, their households spend 2,980 PHP peer week and earn 6,951PHP as income. 23% of the health workers said that they had refrained from seeking medical treatment in the past 12 months because of financial constraints, which is again similar to the general client population.

Table S1 – Demographic characteristics of CHW and other clients

	Regular clients		CHWs		Difference	
	Mean	SD	Mean	SD	Mean difference	SE
Age [20-76]	45.21	(9.89)	42.78	(8.57)	-2.43 *	(1.67)
Years of education [0 - 19]	9.6	(2.88)	10.78	(2.32)	1.19 **	(0.49)
Household size [1-17]	5.46	(2.24)	5.25	(1.63)	-0.214	(0.38)
Married [0/1]	0.69	(0.46)	0.69	(0.47)	0.005	(0.08)
Subjective health rating [0-10]	7.33	(1.94)	7.44	(1.75)	0.110	(0.32)
Weekly hh expenditures in PHP [3-16828]	2,971	(1,842)	2,980	(1925)	-9.41	(312.8)
Weekly hh income in PHP [5-47000]	6,398	(4777)	6,951	-(4147)	-553.07	(806.8)
Did not seek medical treatment because of financial constraints [0/1]	0.2	(0.40)	0.23	(0.48)	0.029	(0.069)

Notes: Demographic profiles of regular clients (N=1028) and CHW (N=36). P-value: * p≤0.1, ** p≤0.05, *** p≤0.01

S2.2 Data collection

Figure S1 illustrates the intervention process and gives an overview of the employed research instruments. The data was collected in two survey waves in February 2014 and April 2015 covering a time span of more than a year. The *baseline survey* was conducted with a subsample of 792 respondents. Respondents were randomly drawn among the eligible clients from the 70 selected centers. The main questionnaire included sections on the respondent's utilization of KDCI services, her health constitution, health behavior, health knowledge, and different socio-demographic and household characteristics.

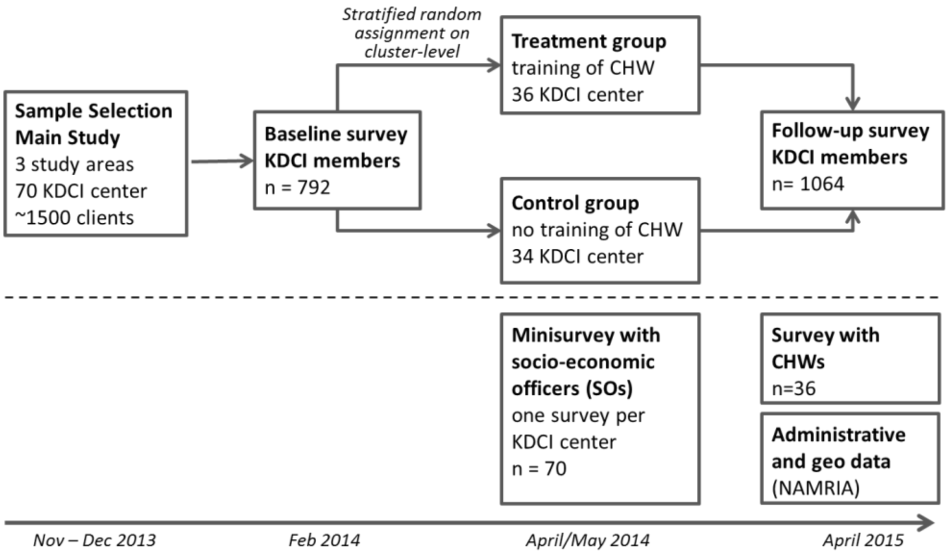

Figure S1 - Research process and instruments

The sample of the follow-up survey, which was drawn from the same client pool as the baseline survey, was restricted to persons who were KDCI clients at the time of the intervention or who became clients within three months after the start of the intervention to ensure that all respondents were exposed to the intervention for a sufficiently long time. Data about the locations of health facilities was provided to us by the National Mapping and Resource Information Authority (NAMRIA) in the Philippines.

S2.3 Details on Measurement

The transfer of health knowledge and information was measured with three indicators. The first one captures whether respondents were informed about the services offered as part of the KDCI health program in general. The second indicator is a health knowledge index which is based on 28 questions about various health topics. The measure was dichotomized at the median to make it comparable to the other binary measures. The final indicator measures if respondents learned new information about disease threats in the past 6 months.

As second category, changes in health monitoring are considered, which was assessed with four indicators. The survey instrument measured if the respondents underwent a medical examination in the last 12 months either offered as part of the health program of the partner organization or by another provider, if they had their blood pressure measured in the past 3 months (an activity which can be performed by the CHWs), if they had a personal health care provider, i.e. a health professional who

knows them and their disease history well, and if they had a personal health insurance at the time of the survey.

The final outcome category considers the level of social support in the communities, which was measured with three indicators. The first and second indicators capture if the respondent knew a contact person in her general social network (circle of family, friends, and acquaintances) or within her KDCI center to ask for advice or help if she had a health concern. For the third indicator, respondents were subsequently asked if someone among their friends or acquaintances criticizes them or gives them advices on their health behavior, actively shares information about health-related topics with them, encourages them to regularly undergo a routine check-up, and encourages them to seek help from a health professional if they are sick (encouragement by peers). The indicator was coded one if respondents replied yes to at least 2 of the 4 sub-questions (median).

All indicators were aggregated over the different dimensions using two procedures: Indicators were either summed up directly (additive approach) or weighted based on empirical weights calculated by employing principal component analysis [50]. Both the additive and weighted outcome measures were normalized to a range from 0 to 1. The aggregations allow us to gain an intuition of the overall impact of the intervention and to perform heterogeneity analyses.

Please note that both aggregation forms are arbitrary and not guided by theoretical considerations (e.g. if the weights were to depend on the impact of the single indicators on a greater outcome, such as a person's health condition). Therefore, caution in the interpretation of the aggregated outcome measures is warranted.

S2.4 Details on Identification Strategy

We first analyze the impact of the CHW intervention on health outcomes in the communities. Following the potential outcome framework introduced by Rubin (1974), the parameter of interest is the individual treatment effect, i.e. the difference in individual outcomes between the single treatment arms (T,C): $E\left[Y_i^T - Y_i^C\right]$. As it is impossible to observe an individual i in both the treated and the non-treated state at the same time, it is impossible to estimate individual treatment effects. Each individual has two potential outcomes, but only one is observed [52]. To overcome this *'fundamental problem of causal inference'* [53], average differences between individuals in the treatment and control group are considered. Because of the random assignment no other factor influences the selection process allowing us to derive an unbiased estimate of the causal treatment effect.

Even in treatment group centers not all community members made use of the CHW services resulting in incomplete treatment for some individuals. Hence, in the estimation the *intention to treat effects* (ITE) are derived, i.e. the effects of the intervention on all KDCI clients in the treatment group neighborhood, regardless of whether they made use of the CHW's services or not. For the partner organization

the ITE estimate is the parameter of interest taking into consideration that there may always be some clients who do not make use of the services provided by the CHWs. The second part of the evaluation is concerned with the actual mechanism that influence program uptake among the community members.

In the empirical identification, ITE is estimated in a first step by regressing the outcomes Y on actual treatment status T using ordinary least squares (OLS) estimation. Since the CHWs are also part of the sample, a dummy is included in the models which indicates if the respondent is herself a CHW to control for differences in the outcome for the health workers. Furthermore, in the estimation the set of pre-treatment controls C is included.

$$Y_i = \beta_0 + T_i'\beta_1 + CHW_i'\beta_2 + C_i'\beta_3 + \varepsilon_i \tag{1.1}$$

$$\widehat{ITE} = \frac{1}{n}\sum_{i=1}^{N} T_i'\widehat{\beta_1} \tag{1.2}$$

During the experiment, unplanned changes between treatment and control group occurred, which may have led to biases in the estimation. Two-stage-least-squares (2SLS) instrumental variable (IV) estimation is employed to account for these deviations in the experimental procedure. The potentially endogenous actual treatment status T is instrumented with the strictly exogenous original random assignment A in a two-step procedure. Note that this procedure only accounts for non-compliance on center level, the basic unit for the randomization, but does not account for non-compliance on the individual client level.

$$T_i = \alpha_0 + A_i'\alpha_1 + CHW_i'\alpha_2 + C_i'\alpha_3 + \theta_i \tag{2.1}$$

$$Y_i = \gamma_0 + \hat{T}_i'\gamma_1 + CHW_i'\gamma_2 + C_i'\gamma_3 + \varepsilon_i \tag{2.2}$$

Where the estimate of T from the first-stage equation is included in the second-stage, structural equation yielding unbiased estimates of the effect of T on Y. The errors are adjusted for the two-stage estimation procedure. I present both results from the OLS and IV specification in the analysis, even though the IV approach represents the preferred specification as it takes deviations from the experimental protocol into account.

Table S2 shows the first stage estimation results of regressing the actual treatment status on the original treatment status. As there were only few deviations from the experimental protocol, the two

variables are highly correlated making the original treatment status a relevant instrument. Strictly speaking, the results derived from the IV estimation represent local average treatment effects (LATE) given that compliance with the treatment was not guaranteed (comparable to an encouragement design) and given that the CHW's decision to comply or not may at the same time be correlated with the potential intervention outcome. However, non-compliance occurred only in very few cases (<5% of all centers) and the IV estimates strongly resemble the OLS estimates suggesting that the estimates are generalizable to the wider population of centers from which the sample was drawn.

Table S2 - 2SLS first stage estimation: Original and actual treatment status

	Outcome: Actual treatment status	
**Original treatment status**	0.915 ***	[0.044]
**Exogenous controls**		
CHW dummy (direct effect)	0.088 **	[0.041]
Neighborhood: Masinag	-0.118 ***	[0.064]
Neighborhood: Batasan	-0.105 ***	[0.095]
Years of education	-0.002	[0.002]
Cognitive	0.003	[0.003]
Age	0.001	[0.001]
Household size	0.001	[0.001]
Children	0.005	[0.003]
Married	-0.036 **	[0.017]
Distance to next health facility	-0.023	[0.027]
Number of hospitals in 2km range	0.001	[0.005]
Number of clinics in 2km range	0.006	[0.012]
Constant	0.096 **	[0.066]
Observations	1056	
Kleibergen-Paap rk Wald F statistic	415.703	

Notes: First stage IV coefficients in cells, robust standard errors in brackets. P-value: * p≤0.1, ** p≤0.05, *** p≤0.01.

S2.5 Ethical Considerations

The experimental procedure of randomly excluding communities from the potential benefits of the CHW intervention may appear questionable from an ethical standpoint [52,54,55]. Several reasons justify the chosen research: First, the considered CHW intervention was in an early implementation phase and had not yet been proven to be beneficial. Lessons learned from the evaluation can support program improvements and hence benefit the entire KDCI client population in the future. Second, due to capacity constraints, it was not planned by KDCI to introduce the program in any of the

selected treatment group centers. Only because of the evaluation, KDCI decided to extend the program and to conduct additional trainings in the treatment centers on top of the regular training schedule. The intervention was hence not unnecessarily refused to communities which would have otherwise benefited from it. After the follow-up survey in April 2015, the program was gradually phased-in in all control group centers.

The training itself and the implementation of the program were conducted following the regular KDCI procedures, which were, like this study, approved by the *KDCI board of trustees* (including clients of the organization). At no time did the researchers interfere with these procedures. It is worth mentioning that the use of the CHW services by the community members was fully voluntary and clients were able to refuse the services. The evaluation followed common guidelines in social science research [52,56]. Respondents were informed about the content of the study, but they were not informed about the RCT and its purpose to avoid adaptation effects. Prior to each interview, individual informed consent was taken (consent form in Figure A1 in the appendix).

S2.6 Power Analysis

Prior to the survey, a power analysis was performed to determine the required sample size for the follow-up survey. As the randomization was not performed on the individual, but the cluster level, additional factors were taken into consideration that may potentially affect the statistical power: First, the intra-cluster correlation (ICC = 0.05) was calculated based on 6 baseline characteristics that were meaningful with regard to selected evaluation outcomes (subjective health, possession of personal health insurance, utilization of check-up in past 12 months, use of hospital services, felt social support, and health knowledge). Second, the number of clusters (k=70) was pre-determined. Third, the coefficient of variation of cluster sizes (0.4) was calculated based on the standard deviation in size divided by the average size of the actual KDCI centers. The user-written Stata clustersampsi command was used for the power calculation yielding for a detectable difference of 10%, a signif-icance-level of 5%, and a power-level of at least 80% [57]. Standard deviation was assumed to be 0.5 in treatment and control group which is reasonable as most outcomes are binary. The power analysis resulted in a minimum required sample size of 420 respondents per treatment arm and a minimum average cluster size of 6. Both conditions were fulfilled in the sampling.

S2.7 Randomization and Balance Check

Compliance with the experimental procedures, such as the randomized assignment, is critical for the unbiased estimation of intervention effects. In most social science applications a deterministic assignment of an intervention is impossible as participants' behavior cannot be fully controlled for [55].

This section tests if systematic deviations from the experimental protocol occurred. I check for contamination of the treatment and control group, imbalances in the covariate distribution between treatment and control group, spill-over effects, and sample attrition.

Contamination refers to unplanned changes of cluster units between treatment and control group because of non-compliance with the experimental protocol. In four treatment group center, CHWs resigned from KDCI and their CHW function within the one-year evaluation period leaving their centers only partly treated. Fortunately, all drop-outs occurred either shortly after the training or shortly before the follow-up survey in April 2015 allowing us to make a clear separation: In three centers CHWs dropped out later than February 2015. These centers were considered as fully treated in the evaluation. In one center, the CHW resigned 3 months after the training and never actively pursued her activities as health worker. I decided to consider this center as untreated in the empirical analysis. On the other hand, also few shifts from the control to treatment group occurred: In two control group centers clients attended the CHW training sessions and hence contaminated the treatment group. I account for these deviations in the estimation strategy, which will be presented in the next sub-section. Overall, changes in treatment status occurred in less than 5% of all centers.

Second, I check for *imbalances* in the distribution of covariates and outcome variables between treatment arms, which may confound the impact evaluation. Imbalances can either occur initially if the original randomization did not create balanced treatment arms, for example because of a too small numbers of cluster units, or due to systematic changes in treatment status (contamination) after the assignment. To test if the initial randomization created a balanced sample 20 outcome and background characteristics measured in the baseline survey are regressed on the *original treatment status*. If the sample was balanced, there should be no major differences between the treatment arms. After this, the test described above is repeated using the *actual treatment status* instead of the original status on the right-hand side of the equation to account for the consequences of the contamination. Results of the estimation are reported in Table S3. No significant differences (a=5%) between treatment and control group are observable neither for the original nor for the actual treatment status suggesting that the balance property is fulfilled in this sample.

Table S3 - Balance check: Regressions on original and actual treatment status

**Outcomes** (range in brackets)	Original treatment status		Actual treatment status		N	R^2
Health knowledge [1-10]	0.006	[0.137]			788	0.002
			-0.062	[0.135]	788	0.002
Fruit consumption [0/1]	-0.071	[0.190]			792	0.007
			-0.069	[0.189]	792	0.007
Exercising [0/1]	-0.049	[0.036]			792	0.009
			-0.055	[0.037]	792	0.010
Personal health insurance [0/1]	0.019	[0.045]			792	0.008
			0.003	[0.045]	792	0.008
Having soap near toilet [0/1]	0.042	[0.032]			792	0.005

			0.025	[0.034]	792	0.003
Underwent KDCI routine check-up in	0.007	[0.030]			792	0.004
past year [0/1]			0.016	[0.030]	792	0.004
Underwent routine check-up	-0.017	[0.050]			792	0.006
By other organization [0/1]			-0.05	[0.051]	792	0.008
Sought help after experiencing disease	0.034	[0.045]			737	0.007
symptoms [0/1]			0.039	[0.046]	737	0.007
Hospitalization in past year [0/1]	-0.028	[0.041]			783	0.015
			-0.043	[0.041]	783	0.016
Knowing a person to ask for help if	-0.02	[0.034]			792	0.004
sick [0/1]			-0.047	[0.033]	792	0.006
Having a personal health care provider	-0.051	[0.050]			788	0.015
[0/1]			-0.086	[0.048]	788	0.019
Number of disease symptoms in past 3	0.29	[0.239]			792	0.014
months [0-∞]			0.322	[0.256]	792	0.014
Subjective health rating [0-10]	-0.166	[0.156]			792	0.008
			-0.205	[0.156]	792	0.009
Household income per capita [0-∞]	104.586	[93.855]			786	0.011
			98.996	[95.788]	786	0.011
Experiencing financial constraints in	0.011	[0.050]			792	0.015
health behaviour [0/1]			0.016	[0.051]	792	0.015
Household size [0 - ∞]	-0.17	[0.159]			792	0.002
			-0.288	[0.161]	792	0.005
Distance next hospital [a] [0 - ∞]	0.169	[0.264]			1064	0.292
			0.319	[0.264]	1064	0.306
Distance next health center [a] [0 - ∞]	-0.0121	[0.181]			1064	0.188
			0.0384	[0.170]	1064	0.189
# hospitals in 5km range [a] [0 - ∞]	0.862	[1.129]			1064	0.634
			1.156	[1.122]	1064	0.637
# health centers in 5km range [a] [0 - ∞]	1.213	[1.645]			1064	0.634
			1.321	[1.626]	1064	0.634

Notes: Linear regression coefficients in cells and standard errors in brackets. Standard errors are clustered on center level (m=70). Original treatment status and actual treatment status are dummy variables taking the value one if respondent lived in a center in the (original or actual) treatment group. [a] These analyses were performed using the follow-up data, assuming that the locations of health facilities have not changed in the evaluation period. P-value: * p≤0.1, ** p≤0.05, *** p≤0.01

Spill-overs between treatment and control group are another threat for the identification of treatment effects. Spill-overs can occur for example if KDCI clients from control group centers benefited of the existence of a CHW in a neighboring center. I test for spill-overs in the main models by including a variable counting the number of CHW in a range of 1km around the respondent's home (see Table S4). The coefficient of this variable is not significant in any of the considered models. The results are similar if I use a dummy indicating whether or not a client lives in close distance (<1km) to another treatment group center or a variable that measures the distance to the next CHW.

Table S4 – Check for spill-overs

	Additive outcome measure			Weighted outcome measure		
	OLS	OLS	2SLS	OLS	OLS	2SLS
# of (other) treatment group center in	-0.002	-0.004	-0.004	-0.003	-0.005	-0.005
1km range around respondent's home	[0.004]	[0.004]	[0.004]	[0.004]	[0.004]	[0.004]
Actual treatment		0.035**	0.041***		0.035**	0.042**
		[0.011]	[0.012]		[0.013]	[0.014]
Constant	0.324***	0.303***	0.299***	0.245***	0.223***	0.219***
	[0.043]	[0.043]	[0.043]	[0.048]	[0.049]	[0.049]
Observations	1056	1056	1056	1056	1056	1056
Adjusted R^2	0.035	0.043	0.043	0.061	0.067	0.067
AIC	-609.333	-616.974	.	-369.298	-374.688	.

Notes: OLS and 2SLS regression coefficients in cells, standard errors in brackets. Standard errors are clustered on center level (m=70). Additional controls included, but not displayed: Neighborhood fixed effects, years of education, age, household size, marital status, distance to next health facility, number of hospitals and health clinics in 2 and 5km range around respondent's home. P-value: * p≤0.1, ** p≤0.05, *** p≤0.01

Furthermore, CHWs were directly asked in additional interviews whether they were consulted by a member from another center, which happened only in one single case. Although with this approach not all possible transmission channels may be captured, the findings suggest that spill-overs from treatment to control group centers were not a major issue in this evaluation, especially given that exchanges between clients from different centers are rather rare as most centers are not located in close proximity.

Few clients who participated in the baseline sample resigned from Kasagana-Ka in the period between the baseline and follow-up survey. Such *attrition* can be harmful for the identification, if it happens in a systematic way, i.e. if the drop-out is related to the treatment status, which may cause a self-selection bias in the estimation [58]. To test for systematic attrition, drop-out from the organization was regressed on original and actual treatment status (Table S5). None of the models indicate a significant correlation between resignation and treatment status indicating that attrition did not occur because of the intervention. Still, the observed drop-out may be correlated with other client characteristics and hence leave us with a different sample than in the baseline survey. The sample selection was restricted in the follow-up to individuals who were clients of KDCI at least for 9 months at the time of the survey. This ensures that the respondents were exposed to the intervention for a sufficiently long period of time. The majority of the respondents in the follow-up belong to the more stable, long-term core client population of KDCI (average membership duration in the follow-up is 45 months).

Table S5 – Check for attrition by treatment group status

	Attrition	
	- 1 -	- 2 -

Original treatment status	-0.015	
	[0.049]	
Actual treatment status		-0.024
		[0.049]
Observations	792	792
Adjusted R^2	0.001	0.001
AIC	1016.10	1015.79

Notes: OLS regression coefficients in cells, standard errors in brackets. Standard errors are clustered on center level (m=70). P-value: * p≤0.1, ** p≤0.05, *** p≤0.01

S2.8 Details on Social Network Data Collection

To capture the social networks of KDCI members in the centers, respondents answered to a social network questionnaire with four questions about their relationship to all other center members: “I am interested in your relationship to the other center members. [1] Could you please tell me who of the members on this list you consider to be your personal friend? [2] And who of the members do you meet at least once every week besides the regular center meetings for at least 15 minutes continuously? [3] Who of the members do you consider to be one of your 5 best friends? [4] To whom of the members do you speak about your personal, intimate problems, such as your health situation?”

The resulting complete *sociometric network data* provides not only information about individual relationships in each center, but also about the structural position of clients in the entire microfinance group network [41,45]. The relationships between the clients were saved in adjacency matrices. They can be depicted in form of network graphs as a set of notes, which are connected through (weak or strong) ties/edges. Figure S2 shows the geographical location of the clients’ homes in one exemplary microfinance group and the corresponding social network of clients.

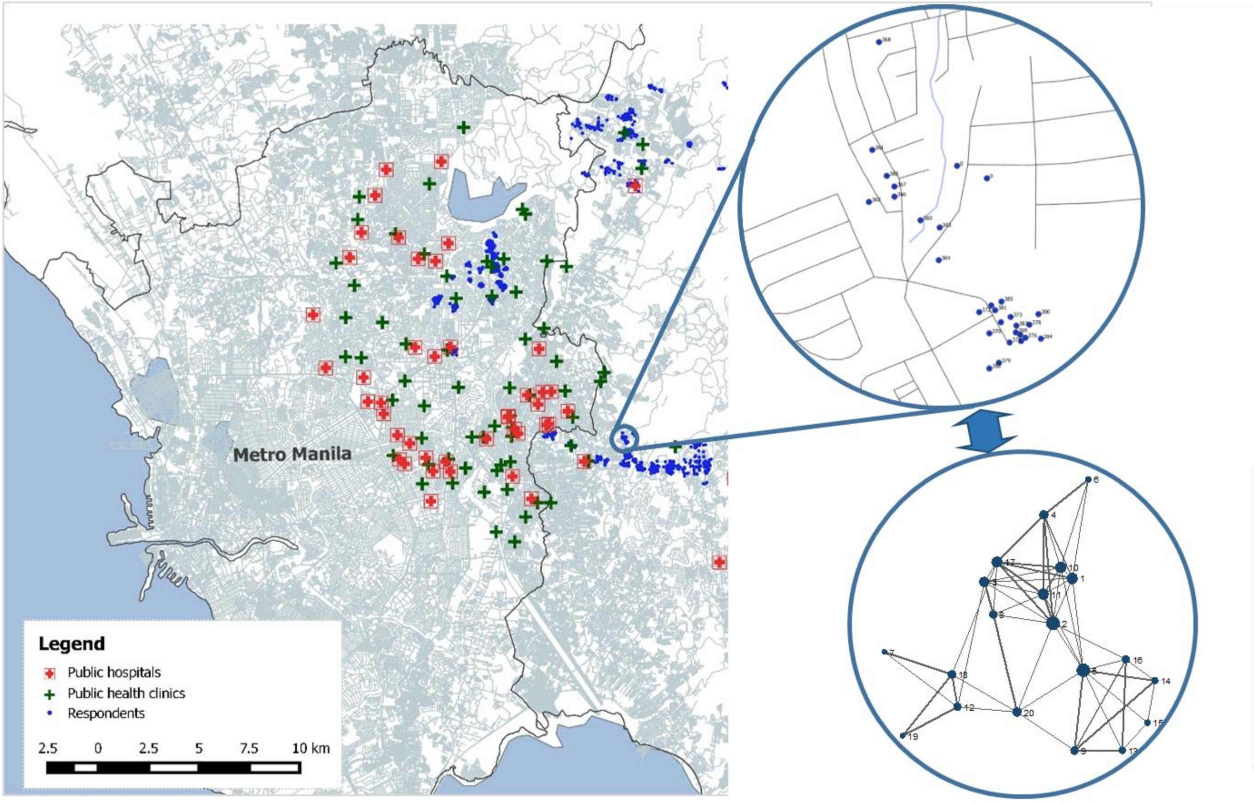

Figure S2 – Illustration of social network data collection

S3. Sensitivity Analyses and Further Results

S3.1 Descriptives

The intervention started in April 2015 in 37 centers (36 centers after accounting for contamination). The assignment was stratified across the three main geographical areas resulting in an almost equal distribution of respondents across the neighborhoods ranging from 49% in the first study area ('Montalban') to 58.4% in the third ('Batasan'). In total, 570 respondents (including CHWs) or 53.6% of the sample were members of original treatment group centers (577 or 54.2% after accounting for contamination). Table S6 shows the distribution of treatment and control group by study areas. Table S7 shows additional summary statistics on background characteristics in the three study areas. The last area Batasan consists of mostly urban communities, whereas the first two represent rural or peri-urban communities, which is reflected in a worse access to the public health infrastructure.

Table S6 - Original treatment status by study areas

Original assignment	Study area Montalban	Masinag	Batasan	Total
Control	203<br>51.0%	154<br>45.7%	137<br>41.6%	494<br>46.4%
Treatment	195<br>49%	183<br>54.3%	192<br>58.4%	570<br>53.6%
Total	398<br>100%	337<br>100%	329<br>100%	1064<br>100%

Table S7 – Additional summary statistics on background characteristics in neighborhoods

	Masinag		Montalban		Batasan	
**Control variables**						
Years of education [0 - 19]	9.99	(2.60)	9.21	(3.20)	9.66	(2.76)
Age [20-76]	44.86	(10.07)	43.55	(9.99)	47.08	(9.13)
Household size [1-17]	5.43	(2.41)	5.4	(2.07)	5.55	(2.13)
Married [0/1]	0.65	(0.48)	0.69	(0.47)	0.74	(0.44)
Distance to health facility in km[0.02-3.77]	1.04	(0.42)	1.1	(1.01)	0.45	(0.22)
# hospitals in 2km range [a] [0 - 13]	1.62	(2.97)	0.29	(0.45)	1.21	(1.32)
# health centers in 2km range [a] [0 - 11]	2	(2.09)	1.69	(1.13)	6.62	(1.34)
# hospitals in 5km range [a] [0 - 34]	11.61	(5.62)	0.82	(0.39)	14.56	(4.99)
# health centers in 5km range [a] [0 - 43]	14.48	(9.22)	4.58	(1.82)	26.32	(5.69)
**Other variables**						
Subjective health rating [0-10]	7.18	(1.91)	7.3	(1.96)	7.57	(1.91)
Weekly hh expenditures in PHP [3-16828]	3118.28	(2042.47)	2,651.63	(1694.96)	3,122.13	(1696.04)
Weekly hh income in PHP [5-47000]	6,909.1	(4980.2)	6,028.9	(5203.1)	6,223.7	(3893.9)
Did not seek medical treatment because of financial constraints [0/1]	0.22	(0.41)	0.2	(0.40)	0.21	(0.41)

Figure S3 and S4 show the distribution of the key composite outcomes (additive and weighted) in the sample and the distribution of the CHW's indegree in the community. The distribution of the two outcome variables approximately follows a bell shape, with most of the mass being located at the center of the variable range.

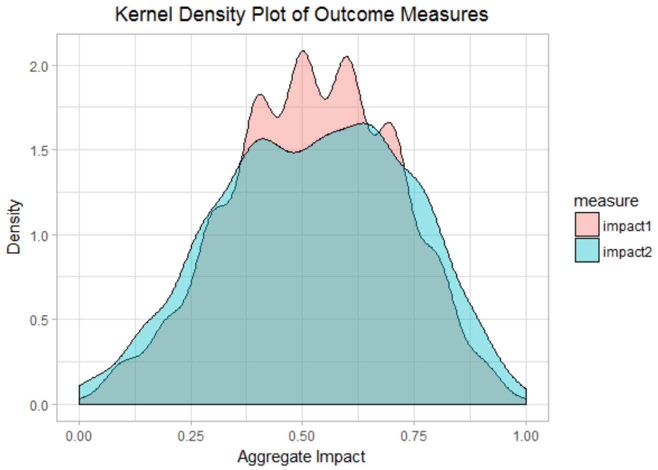

Figure S3 - Distribution of aggregate outcome measures

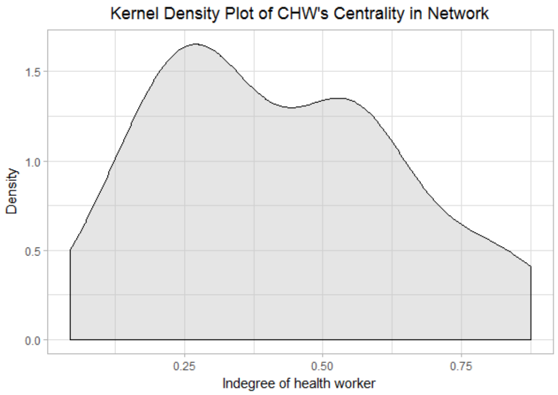

Figure S4 - Distribution of CHW's indegree in treatment group centers

S3.2 Impact Estimation for Separate Outcome Indicators

Table S8 shows the impact estimation (main text section 4.3) for the separate outcome indicators using both OLS and 2SLS models. According to these, the CHWs were successful in disseminating information about the health program of the partner organization and in improving health monitor-

ing in the communities by raising clients' probability to undergo a check-up, having their blood pressure measured, and having access to a professional health care provider. There is no statistically significant effect on clients' general health knowledge as well as their perceived social support.

Higher levels of health knowledge (both specific related to the program of the partner organization and in general) are observable among the community health workers. These differences in knowledge could represent a direct effect of the intervention on the health workers. However, these effects cannot be interpreted as causal as they may stem from pre-intervention differences between the CHWs and other KDCI clients.

Table S8 – OLS and 2SLS models: Impact estimation for separate outcome indicators

		**Treatment group dummy**		**CHW Dummy**		**Constant**		**Obs**	**Adj R^2**
**A. Information dissemination**									
Info about health program	OLS	0.065+	[0.035]	0.157*	[0.064]	0.111	[0.154]	1057	0.031
	2SLS	0.076*	[0.037]	0.152*	[0.063]	0.104	[0.150]	1057	0.031
General health knowledge	OLS	-0.023	[0.030]	0.154*	[0.073]	-0.459***	[0.102]	1057	0.136
	2SLS	-0.010	[0.032]	0.148*	[0.071]	-0.468***	[0.102]	1057	0.135
Info about disease threats	OLS	0.013	[0.029]	0.045	[0.087]	-0.028	[0.159]	1057	0.009
	2SLS	-0.008	[0.031]	0.055	[0.087]	-0.014	[0.156]	1057	0.009
**B. Health monitoring**									
Any check-up last year	OLS	0.067+	[0.036]	0.159*	[0.075]	0.012	[0.131]	1057	0.036
	2SLS	0.106**	[0.038]	0.140+	[0.074]	-0.014	[0.127]	1057	0.035
Regular BP checks	OLS	0.085**	[0.030]	-0.073	[0.077]	0.359**	[0.136]	1057	0.011
	2SLS	0.096**	[0.032]	-0.078	[0.076]	0.352**	[0.130]	1057	0.010
Access to health care provider	OLS	0.088**	[0.029]	0.003	[0.081]	0.299*	[0.138]	1057	0.018
	2SLS	0.072*	[0.032]	0.010	[0.080]	0.309*	[0.136]	1057	0.018
Health insurance	OLS	0.019	[0.026]	-0.015	[0.065]	0.053	[0.109]	1057	0.009
	2SLS	0.023	[0.027]	-0.017	[0.063]	0.050	[0.110]	1057	0.009
**C. Social support**									
Contact person in general	OLS	0.005	[0.027]	-0.021	[0.075]	0.775***	[0.089]	1057	-0.003
	2SLS	0.008	[0.029]	-0.022	[0.073]	0.774***	[0.087]	1057	-0.003
Contact person in center	OLS	0.012	[0.034]	-0.136+	[0.073]	0.321**	[0.111]	1057	-0.002
	2SLS	-0.012	[0.022]	0.026	[0.054]	0.960***	[0.077]	1055	-0.003
Encouragement by peers	OLS	0.002	[0.026]	0.016	[0.056]	0.890***	[0.085]	1057	0.008
	2SLS	0.001	[0.028]	0.017	[0.055]	0.891***	[0.087]	1057	0.008

Notes: OLS coefficients in cells, standard errors in brackets. Standard errors are clustered on center level (m=70). P-value: * p≤0.1, ** p≤0.05, *** p≤0.01

S3.3 Impact Heterogeneity across Treatment Group Centers: The Role of Acceptance

Overall, the intervention had a positive impact in the communities: The CHWs were able to significantly raise awareness about the services offered by the partner organization and to promote health monitoring. On the other hand, there is no impact on broader outcomes, such as general health knowledge and social support, which suggests that the CHW program has not yet achieved all of its target objectives. One reason for the restricted impact may be the overall low level of program utilization and awareness. Also, great variation in program acceptance across neighborhoods is observable. While in some centers none of the clients made use of the health workers services (4 centers in total), in one center almost all members (83.3%) said that they consulted the CHW in the past 12 months. It seems as if the initiative was successful in attracting clients' interest only in some areas.

If the aggregate outcome measures are analyzed separately for the different treatment group centers, a substantial heterogeneity is observable. While some centers perform exceptionally well, others show much lower levels on the aggregate outcome suggesting that some centers may have benefited over-proportionally from the program. Figure S5 depicts differences in the additive aggregate outcome between treatment group centers compared to the control group level (red bar). The figure distinguishes further between treatment group neighborhoods with high and low levels of acceptance based on the overall awareness and uptake of CHW services in the community (wider acceptance definition). In centers with high acceptance, the majority of interviewed center members either knew the name of the local health worker or made use of her services.

Linking acceptance and the aggregate program outcome reveals a clear relationship. Centers with higher acceptance score on average higher on the aggregate outcome. Although this evidence cannot be interpreted as causal, it is intuitively plausible: For the intervention to have an impact it is necessary to raise community members' awareness of it and to encourage uptake. This conclusion is supported if differences in the aggregate outcome on individual level are considered. Figure S6 compares the distribution of the aggregate outcome measure between individuals in the control group and individuals in treatment group centers who express low and high acceptance levels according to the wider acceptance definition. Clients from treatment group centers who have either used the CHW services or are at least aware of her activities show on average a higher aggregate outcome compared to clients with low acceptance or clients living in control group centers ($p \leq 0.01$, t-test). In contrast, respondents with low levels of acceptance, although exposed to the intervention, do not show significantly higher health outcome levels as compared to respondents living in control group centers ($p > 0.1$, t-test, assuming identical distributions for both tests). The results suggest that sufficient anchoring in the communities is a key condition for the program to have a positive impact.

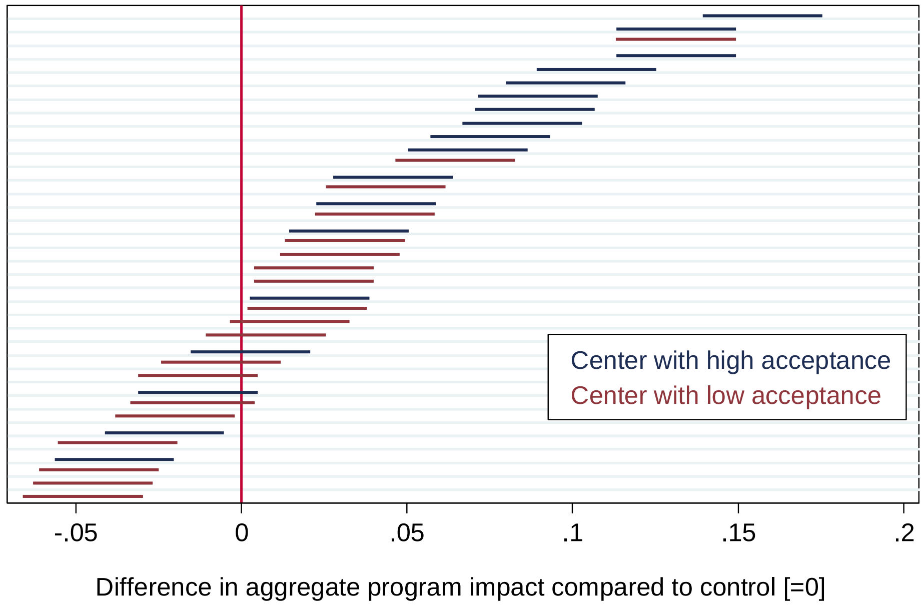

Figure S5 - Heterogeneity in program impacts across treatment group neighborhoods

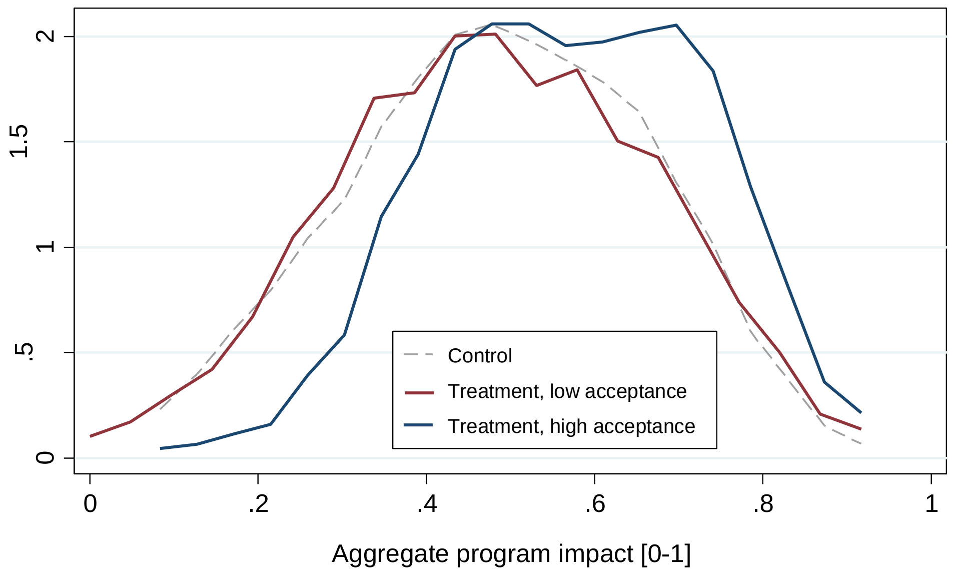

Figure S6 - Heterogeneity in program impacts between individuals

S3.4 Social Influence: Full Models

Table S9 shows the full social influence models (main text section 4.4) including the full set of controls. Apart from the social network indicators, none of the included controls consistently predicts the program outreach and impact indicators.

Table S9 – Logit and OLS models: Drivers of program acceptance and impact (all controls displayed)

	Program awareness Logit		Program utilization Logit		Additive outcome OLS	
	- 1 -	- 2 -	- 3 -	- 4 -	- 5 -	- 6 -
**Social network indicators**						
Indegree centrality of CHW [0-1]	0.561**	0.198	0.729***	0.357*	0.151*	0.068
	[0.208]	[0.222]	[0.151]	[0.155]	[0.064]	[0.071]
Weak relationship with CHW [0/1]		0.186***		0.159**		0.070**
		[0.050]		[0.050]		[0.024]
Strong relationship with CHW [0/1]		0.333***		0.354***		0.062*
		[0.051]		[0.029]		[0.026]
**Other controls**						
Years of education	0.007	0.009	-0.007	-0.005	0.009**	0.009**
	[0.008]	[0.007]	[0.006]	[0.006]	[0.003]	[0.003]
Cognitive abilities	0.023	0.016	0.008	0.002	0.010+	0.008
	[0.019]	[0.018]	[0.011]	[0.010]	[0.005]	[0.005]
Age	0.003	0.002	0.002	0.002	0.002+	0.002
	[0.002]	[0.002]	[0.002]	[0.002]	[0.001]	[0.001]
Household size	-0.007	-0.011	-0.024	-0.031+	0.002	0.002
	[0.016]	[0.016]	[0.017]	[0.017]	[0.005]	[0.005]
Number of children	0.016	0.026	0.031	0.043*	0.006	0.007
	[0.022]	[0.023]	[0.021]	[0.019]	[0.006]	[0.007]
Marital status	0.020	0.005	0.041	0.022	-0.006	-0.008
	[0.046]	[0.050]	[0.046]	[0.049]	[0.013]	[0.013]
Distance to next health center	-0.005	-0.016	0.064	0.054	-0.011	-0.015
	[0.049]	[0.046]	[0.046]	[0.036]	[0.014]	[0.015]
Number of hospitals in 2km range	-0.007	-0.002	-0.010	-0.007	-0.004	-0.003
	[0.012]	[0.012]	[0.011]	[0.012]	[0.003]	[0.003]
Number of clinics in 2km range	0.013	0.010	0.013	0.010	-0.002	-0.003
	[0.017]	[0.017]	[0.013]	[0.015]	[0.004]	[0.005]
Density	-0.002	-0.000	-0.001	0.002	-0.002	-0.001
	[0.003]	[0.004]	[0.003]	[0.003]	[0.002]	[0.002]
Network size	-0.636	-0.273	-0.760*	-0.297	-0.360+	-0.300
	[0.390]	[0.395]	[0.308]	[0.329]	[0.193]	[0.184]
Distance to CHW home	-7.427	-6.068	-3.245	-2.932	2.802	3.261
	[5.272]	[5.732]	[6.574]	[5.820]	[1.899]	[2.024]
Observations	538	538	538	538	538	538
Pseudo/adjusted R^2	0.032	0.091	0.083	0.219	0.018	0.043
AIC	749.261	709.436	583.706	505.986	-308.984	-320.863

Notes: Marginal effects and OLS coefficients in cells, standard errors in brackets. Standard errors are clustered on center level (m=70). All controls included in the models, but not displayed: years of education, cognitive abilities, age household size, number of children, marital status, neighborhood dummies, distance to next health facility, number of clinics and hospitals in 2km range. P-value: * p≤0.1, ** p≤0.05, *** p≤0.01

S3.5 Sensitivity Checks

The social influence estimates are not based on experimental data and may hence be prone to endogeneity problems. The following tables present sensitivity checks, which use the second weighted composite health index as alternative outcome (Table S10), use a tie strength measure instead of the binary relationship indicator (no vs. weak vs. strong relationship), which sums up the individual relationship questions (meeting, friendship, close friendship, and sharing of intimate information) to a tie strength index, which ranges from 0-4 (Table S11), or add an additional network variable that measures the proportion of friends who are connected with the CHW (Table S12). The results of these additional tests widely confirm the findings in the main analysis.

Table S10 – OLS models: The second aggregate impact measure as alternative outcome

	Weighted outcome OLS	
	- 1 -	- 2 -
**Social network indicators**		
Indegree centrality of CHW[0-1]	0.160*	0.079
	[0.074]	[0.079]
Weak tie with CHW [0/1]		0.072*
		[0.027]
Strong tie with CHW [0/1]		0.056+
		[0.031]
Observations	538	538
Adjusted R^2	0.036	0.055
AIC	-194.639	-203.273

Notes: OLS coefficients in cells, standard errors in brackets. Standard errors are clustered on center level (m=70). All controls included in the models, but not displayed: years of education, cognitive abilities, age household size, number of children, marital status, neighborhood dummies, distance to next health facility, number of clinics and hospitals in 2km range. P-value: * p≤0.1, ** p≤0.05, *** p≤0.01

Table S11 – Logit and OLS models: Tie strength as alternative relationship measure

	Program awareness Logit		Program utilization Logit		Additive outcome OLS	
	- 1 -	- 2 -	- 3 -	- 4 -	- 5 -	- 6 -
**Social network indicators**						
Indegree centrality of CHW[0-1]	0.561**	0.390+	0.729***	0.624***	0.160*	0.130+
	[0.208]	[0.210]	[0.151]	[0.161]	[0.074]	[0.072]
Strength of tie to CHW [0-4]		0.119***		0.095***		0.018*
		[0.021]		[0.011]		[0.009]
Observations	538	531	538	531	538	531
Pseudo/adjusted R^2	0.032	0.096	0.083	0.183	0.036	0.046
AIC	749.261	695.903	583.706	522.474	-194.639	-194.814

Notes: Marginal effects and OLS coefficients in cells, standard errors in brackets. Standard errors are clustered on center level (m=70). All controls included in the models, but not displayed: years of education, cognitive abilities, age household size, number of children, marital status, neighborhood dummies, distance to next health facility, number of clinics and hospitals in 2km range. P-value: * p≤0.1, ** p≤0.05, *** p≤0.01

Table S12 – Logit and OLS models: Effects of peers' connectedness

	Program awareness Logit		Program utilization Logit		Additive outcome OLS	
	- 1 -	- 2 -	- 3 -	- 4 -	- 5 -	- 6 -
**Social network indicators**						
Indegree centrality of CHW[0-1]	0.228	0.231	0.259	0.343+	0.112	0.340*
	[0.251]	[0.342]	[0.176]	[0.190]	[0.077]	[0.136]
Weak tie with CHW [0/1]	0.184***	-	0.161**	-	0.067**	-
	[0.052]		[0.050]		[0.024]	
Strong tie with CHW [0/1]	0.331***	-	0.356***	-	0.060*	-
	[0.052]		[0.029]		[0.026]	
Proportion of friends connected	-0.043	-0.126	0.151+	-0.061	-0.061	-0.171***
with CHW [0-1]	[0.096]	[0.116]	[0.084]	[0.072]	[0.040]	[0.046]
Observations	536	246	536	246	536	246
Pseudo/adjusted R^2	0.091	0.076	0.222	0.118	0.045	0.052
AIC	709.885	310.475	505.020	154.008	-323.868	-136.725

Notes: Marginal effects and OLS coefficients in cells, standard errors in brackets. Standard errors are clustered on center level (m=70). All controls included in the models, but not displayed: years of education, cognitive abilities, age household size, number of children, marital status, neighborhood dummies, distance to next health facility, number of clinics and hospitals in 2km range. P-value: * p≤0.1, ** p≤0.05, *** p≤0.01

S3.6 Instrumenting Relationship Status with Social Distance Measures

To further test the robustness of the findings, 2SLS instrumental variable regressions are used. The relationship status as identified key network variable is instrumented with plausibly exogenous measures of social distance. These are expected to influence the outcomes of interest only by affecting the likelihood for the existence of a tie with the health worker, but through no other channel (exogeneity assumption). The social distance measures capture the difference in age, cognitive abilities, and wealth between the respondent and the health worker and whether the two were born in the same region of the country. For tractability, the analysis focuses on a simple relationship measure of whether a client had any connection with the CHW or not. All four instruments are relevant for this network indicator as can be inferred from the first stage models (Table S14). The results of the second stage are presented in Table S13 together with various model summaries. Again, all models control for the full set of control variables. The 2SLS models provide support for the previous findings. Having a connection to the health worker, significantly raises the clients' awareness by 29.4% ($p<0.1$), the probability to take-up the program's services by 27.8%, and the aggregate impact by 15.5% and 13.2% ($p<0.1$), respectively, depending on the impact measure used. Please note that these results should be treated with care as the instruments only weakly identify the endogenous regressor (F-Statistics of 7.267) and the models are underpowered due to the small sample size. Nevertheless, the models provide further indication that it is indeed the social networks in the microfinance groups, which are driving some of the impact heterogeneities. Being connected to the health worker is found to have an effect on the clients in terms of both program outreach and effectiveness.

Table S13 – 2SLS models: Social network drivers of program acceptance and impact

	Program awareness	Program utilization	Additive outcome	Weighted outcome
	- 1 -	- 2 -	- 3 -	- 4 -
**Social network indicators**				
Any relationship with CHW	0.294+	0.278*	0.155*	0.132+
	[0.178]	[0.134]	[0.069]	[0.078]
Kleibergen-Paap Wald F statistic	7.267	7.267	7.267	7.267
Hansen J statistic	1.386	2.491	4.469	3.677
P-val Hansen overid. test	0.7088	0.4768	0.2151	0.2985
Observations	538	538	538	538
Adjusted R^2	0.077	0.144	-0.004	0.034
AIC	749.809	555.524	-296.857	-193.591

Notes: Marginal effects and OLS coefficients in cells, standard errors in brackets. Standard errors are clustered on center level (m=37). All controls included in the models, but not displayed: years of education, cognitive abilities, age household size, number of children, marital status, neighborhood dummies, distance to next health facility, number of clinics and hospitals in 2km range. P-value: * p≤0.1, ** p≤0.05, *** p≤0.01

Table S14 – First Stage Regression: Instrumenting Relationship status

	**Any relationship with the CHW**	
**Instruments**		
Difference in age	-0.007**	[0.002]
Difference in cognitive abilities	-0.044*	[0.018]
Difference in wealth	-0.087*	[0.041]
Common birth region	0.105+	[0.068]
**Controls**	-0.007**	[0.002]
Years of education	-0.006	[0.010]
Cognitive abilities	0.013	[0.016]
Age	0.005*	[0.002]
Household size	-0.001	[0.014]
Number of children	-0.012	[0.020]
Marital status	0.02	[0.054]
Distance to next health center	0.107+	[0.061]
Number of hospitals in 2km range	-0.008	[0.018]
Number of clinics in 2km range	0.026	[0.023]
Network density	0.653*	[0.300]
Network size	0.001	[0.004]
Distance to CHW	-10.282	[6.657]
Neighborhood dummy 1	-0.001	[0.121]
Neighborhood dummy 2	-0.002	[0.110]
Constant	0.13	[0.322]
Observations	538	
Adjusted R^2	0.073	
AIC	757.403	

Notes: First stage regression coefficients in cells, standard errors in brackets. Standard errors are clustered on center level (m=37). P-value: * p≤0.1, ** p≤0.05, *** p≤0.01

S3.7 Differences in Outcome Variables by CHW status

Differences in health outcomes between clients who are strongly connected to the CHW and those who are not do not necessarily have to result from the higher awareness and uptake levels, but could also reflect pre-treatment differences resulting from homophilous peer-group formation and selection effects [59]. To test for the existence of pre-treatment differences, Table S15 regresses different outcome indicators measured in the baseline survey on network characteristics (indegree of CHW in network and relationship status). No significant differences are observable between centers with a high and low status CHW and between clients with and without a connection to the health worker. This further suggests that the observed impact heterogeneities are in part driven by the social structures in the microfinance groups, which have a strong influence on whether clients are aware and make use of the CHW intervention.

Table 15 – OLS Models: Baseline outcome indicators regressed on network indicators

	CHW Indegree	Weak tie with CHW	Strong tie with CHW	Obs	Adj R^2
**A. Information dissemination**					
Client is aware of KDCI program	-0.103	-0.077	0.029	241	0.005
	[0.116]	[0.061]	[0.060]		
General health knowledge	0.133	-0.209	-0.235	240	-0.009
	[0.882]	[0.241]	[0.194]		
**B. Health monitoring**					
Any KDCI check-up last year	-0.167	0.095	0.150*	241	0.016
	[0.147]	[0.059]	[0.066]		
Check-up from another organization	0.089	0.026	0.086	240	-0.005
	[0.242]	[0.089]	[0.094]		
Access to health care provider	0.029	0.088	0.068	236	-0.006
	[0.178]	[0.074]	[0.083]		
Health insurance	-0.085	0.034	-0.061	239	-0.005
	[0.222]	[0.079]	[0.075]		
**C. Social support**					
Contact person in general	0.135	-0.118	-0.019	241	0.001
	[0.211]	[0.083]	[0.070]		

Notes: OLS coefficients in cells, standard errors in brackets. Standard errors are clustered on center level (m=37). P-value: * p≤0.1, ** p≤0.05, *** p≤0.01

References

1 Asian Development Bank. Basic Statistics Philippines. Manila: Asian Development Bank; 2018.
2 Bossert TJ. Decentralization of health systems in Ghana, Zambia, Uganda and the Philippines: a comparative analysis of decision space. Health Policy Plan. 2002;17(1):14–31.
3 WHO. Philippines Country Health Profile. Geneva: WHO; 2013.
4 WHO. World Health Statistics 2013. Geneva: WHO; 2013.
5 Solon O, Alabastro-Quimbo S, Panelo CIA. Financing Health Care for Poor Filipinos. Work Pap. 2004;2004–8.
6 Republic of the Philippines Department of Health. Annual Report 2015. Manila; 2015.
7 WHO. Migration of Health Workers. Geneva: WHO; 2014.
8 Lorenzo FME, Galvez-Tan J, Icamina K, Javier L. Nurse migration from a source country perspective: Philippine country case study. Health Serv Res. 2007;42(3 II):1406–18.
9 Leatherman S, Dunford C. Linking health to microfinance to reduce poverty. Bull World Health Organ. 2010;88(6):470–1.
10 Leatherman S, Metcalfe M, Geissler K, Dunford C. Integrating microfinance and health strategies: Examining the evidence to inform policy and practice. Health Policy Plan. 2012;27(2):85–101.
11 Microcredit Summit Campaign. The State of the the Microcredit Summit Campaign Report 2015 [Internet]. 2015. Available from: http://www.microcreditsummit.org/about-the-project3.html
12 Pitt M, Khandker SR, Cartwright J. Empowering women with micro finance: Evidence from Bangladesh. Econ Dev Cult Change. 2006;54(4):791–831.
13 Duflo E. Grandmothers and Granddaughters: Old Age Pensions and Intrahousehold Allocation in South Africa. World Bank Econ Rev. 2003;17(1):1–25.
14 Islam A, Maitra P. Health shocks and consumption smoothing in rural households: Does microcredit have a role to play? J Dev Econ. 2012;97(2):232–43.
15 DeLoach SB, Lamanna E. Measuring the Impact of Microfinance on Child Health Outcomes in Indonesia. World Dev. 2011;39(10):1808–19.
16 Desai J, Tarozzi A. Microcredit, Family Planning Programs, and Contraceptive Behavior: Evidence From a Field Experiment in Ethiopia. Demography. 2011;48(2):749–82.
17 WHO. Declaration of Alma-Ata. International Conference on Primary Health Care, Alma-Ata, USSR, 6-12 Sepember 1978. 1978.
18 Lehman U, Sanders D. Community health workers: What do we know about them? Geneva: WHO; 2007.
19 Reichenbach L. Sustaining Health: The Role of BRAC' s Community Health Volunteers in Bangladesh, Afghanistan, and Uganda. BRAC Res Eval Div. 2011;7(49).
20 Geissler KH, Leatherman S. Providing primary health care through integrated microfinance and health services in Latin America. Soc Sci Med. 2015;132:30–7.
21 Cufino Svitone E, Garfield R, Vasconcelos MI, Araujo Craveiro V. Primary health care lessons from the northeast of Brazil: the Agentes de Saude Program. Rev Panam Salud Publica. 2000;7(5):293–302.
22 Douthwaite M, Ward P. Increasing contraceptive use in rural Pakistan: An evaluation of the Lady Health Worker Programme. Health Policy Plan. 2005;20(2):117–23.
23 Bhutta AZ, Lassi ZS, Pariyo G, Huicho L. Experience of Community Health Workers for

Delivery of Health Related Millennium Development Goals. WHO, Global Health Workforce Alliance Report. Geneva: WHO; 2010.
24 Fisher EB, Coufal MM, Parada H, Robinette JB, Tang PY, Urlaub DM, et al. Peer Support in Health Care and Prevention: Cultural, Organizational, and Dissemination Issues. Annu Rev Public Health. 2014;35(1):363–83.
25 Perry HB, Zulliger R, Rogers MM. Community Health Workers in Low-, Middle-, and High-Income Countries: An Overview of Their History, Recent Evolution, and Current Effectiveness. Annu Rev Public Health. 2014;35(1):399–421.
26 Davis TP, Wetzel C, Hernandez Avilan E, de Mendoza Lopes C, Chase RP, Winch PJ, et al. Reducing child global undernutrition at scale in Sofala Province, Mozambique, using Care Group Volunteers to communicate health messages to mothers. Glob Heal Sci Pract. 2013;1(1):35–51.
27 Taylor CE, Greenough WB. Control of diarrheal diseases. Annu Rev Public Heal. 1989;10:221–44.
28 Luby SP, Agboatwalla M, Painter J, Altaf A, Billhimer WL, Hoekstra RM. Effect of intensive handwashing promotion on childhood diarrhea in high-risk communities in Pakistan: A randomized controlled trial. JAMA J Am Med Assoc. 2004;291(21):2547–54.
29 Luby S, Agboatwalla M, Geikin DR, Painter J, Billhimer W, Altaf a, et al. Effect of handwashing on child health: a randomized controlled trial. Lancet. 2005;366(9481):225–33.
30 Farmer P, Léandre F, Mukherjee J, Gupta R, Tarter L, Kim JY. Community-based treatment of advanced HIV disease: introducing DOT-HAART (directly observed therapy with highly active antiretroviral therapy). Bull World Health Organ. 2001;79(12):1145–51.
31 Wringe A, Cataldo F, Stevenson N, Fakoya A. Delivering comprehensive home-based care programmes for HIV: A review of lessons learned and challenges ahead in the era of antiretroviral therapy. Vol. 25, Health Policy and Planning. 2010. p. 352–62.
32 Chowdhury AM, Chowdhury S, Islam MN, Islam A, Vaughan JP. Control of tuberculosis by community health workers in Bangladesh. Lancet. 1997;350(9072):169–72.
33 Dunford C. Building Better Lives: Sustainable Integration of Microfinance and Education in Child Survival, Reproductive Health, and HIV/AIDS Prevention for the Poorest Entrepreneurs. J Microfinance. 2001;
34 Armin R, St Pierre M, Ahmed A, Haq R. Integration of an Essential Services Package (ESP) in child and reproductive health and family planning with a micro-credit program for poor women: Experience from a pilot project in rural Bangladesh. World Dev. 2001;29:1611–21.
35 Ahmed SM, Petzold M, Kabir ZN, Tomson G. Targeted intervention for the ultra poor in rural Bangladesh: Does it make any difference in their health-seeking behaviour? Soc Sci Med. 2006;63(11):2899–911.
36 Hadi A. Integrating prevention of acute respiratory infections with micro-credit programme. Experience of BRAC, Bangladesh. Public Health. 2002;116:238–44.
37 Dohn AL, Chávez A, Dohn MN, Saturria L, Pimentel C. Changes in health indicators related to health promotion and microcredit programs in the Dominican Republic. Pan Am J Public Heal. 2004;15(3):185–93.
38 Fletcher JM. Peer Effects in Health Behaviors. Encycl Heal Econ. 2014;2:467–72.
39 Meredith J, Robinson J, Walker S, Wydick B. Keeping the doctor away: Experimental evidence on investment in preventative health products. J Dev Econ. 2013;105:196–210.
40 Deri C. Social networks and health service utilization. J Health Econ. 2005;24(6):1076–107.
41 Jackson MO. An overview of social networks and economic applications. Handb Soc Econ. 2011;1:511–85.

42 Devillanova C. Social networks, information and health care utilization: Evidence from undocumented immigrants in Milan. J Health Econ. 2008;27(2):265–86.
43 Granovetter MS. The Strength of Weak Ties. Am J Sociol. 1973;78(6):1360–80.
44 Banerjee A, Chandrasekhar AG, Duflo E, Jackson MO. The diffusion of microfinance. Science (80-). 2013;341(6144):363–70.
45 Marsden P V. Egocentric and sociocentric measures of network centrality. Soc Networks. 2002;24(4):407–22.
46 Jackson MO. An Overview of Social Networks and Economic. Handb Soc Econ. 2010;1–96.
47 Putnam RD. Bowling alone: The collapse and revival of American community. New York: Simon & Schuster; 2000.
48 Shakya HB, Christakis NA, Fowler JH. Social network predictors of latrine ownership. Soc Sci Med. 2015;125:129–38.
49 Perkins JM, Subramanian S V., Christakis NA. Social networks and health: A systematic review of sociocentric network studies in low- and middle-income countries. Soc Sci Med. 2015;125:60–78.
50 Vyas S, Kumaranayake L. Constructing socio-economic status indices: How to use principal components analysis. Health Policy Plan. 2006;21(6):459–68.
51 Rubin DB. Estimating Causal Effects of Treatments in Randomized and Nonrandomized Studies. J Educ Psychol. 1974;66(5):688–701.
52 Duflo E, Glennester R, Kremer M. Using Randomization in Development Economics Research: A Toolkit. In: Rodrik D, Rosenzweig MR, editors. Handbook of Development Economics. 4th ed. 2007. p. 3895–3962.
53 Holland PW. Statistics and Causal Inference. J Am Stat Assoc. 1986;81(396):946–60.
54 Deaton A. Instruments, Randomization, and Learning about Development. J Econ Lit. 2010;48(2):424–55.
55 Barrett CB, Carter MR. The power and pitfalls of experiments in development economics: Some non-random reflections. Appl Econ Perspect Policy. 2010;32(4):515–48.
56 Social Research Association. Ethical Guidelines [Internet]. 2003. Available from: http://the-sra.org.uk/research-ethics/ethics-guidelines/
57 Batistatou E, Roberts C. Sample size and power calculations for trials and quasi-experimental studies with clustering. Stata J. 2014;14(1):159–75.
58 Lee DS. Training, wages, and sample selection: Estimating sharp bounds on treatment effects. Rev Econ Stud. 2009;76(3):1071–102.
59 McPherson M, Smith-Lovin L, Cook JM. Birds of a Feather: Homophily in Social Networks. Annu Rev Sociol. 2001;27(1):415–44.
60 US Department of Health and Human Services. Community Health Workers Evidence-Based Models Toolbox. 2011.
61 Witmer A, Seifer SD, Finocchio L, Leslie J, O'Neil EH. Community health workers: integral members of the health care work force. Am J Public Health. 1995;85(8):1055–8.
62 Perry H, Crigler L, Hodgins S, Advisor T. Developing and Strengthening Community Health Worker Programs at Scale: A Reference Guide for Program Managers and Policy Makers. USAID Report. 2013.
